# Feasibility of high-dose tadalafil and effects on insulin resistance in well-controlled patients with type 2 diabetes (MAKROTAD): a single-centre, double-blind, randomised, placebo-controlled, cross-over phase 2 trial

**DOI:** 10.1016/j.eclinm.2023.101985

**Published:** 2023-05-04

**Authors:** Emanuel Fryk, Vagner Ramon Rodrigues Silva, Marco Bauzá-Thorbrügge, Martin Schmelz, Li-Ming Gan, Lena Strindberg, Per-Anders Jansson

**Affiliations:** a Department of Molecular and Clinical Medicine, Institute of Medicine, Sahlgrenska Academy, University of Gothenburg, SU Sahlgrenska, 413 45 Gothenburg, Sweden; b Department of Neuroscience and Physiology, Institute of Medicine, Sahlgrenska Academy, University of Gothenburg, Box 430, 405 30 Gothenburg, Sweden; c Department of Anesthesiology and Intensive Care Medicine Mannheim, University of Heidelberg, 69117 Heidelberg, Germany; d Ribocure Pharmaceuticals AB, Sweden; e Suzhou Ribo Life Science CO. Ltd, China; f Gothia Forum, Region Västra Götaland, SU Sahlgrenska, 413 45 Gothenburg, Sweden

**Keywords:** Randomised controlled trial, Type 2 diabetes, Phosphodiesterase-5 inhibitors, Metabolic control, Endothelial dysfunction

## Abstract

**Background:**

Phosphodiesterase-5 inhibitors exert positive vascular and metabolic effects in type 2 diabetes (T2D), but the effect on insulin resistance in T2D is unclear.

**Methods:**

This randomised, double blind, placebo-controlled, two-period crossover trial was conducted at Sahlgrenska University Hospital (Gothenburg, Sweden). Men without apparent erectile dysfunction (age 40–70 years) and women (age 55–70 years, post-menopause) diagnosed with T2D between 3 months and 10 years, haemoglobin A1c (HbA1c) < 60 mmol/mol and a body mass index (BMI) 27–40 kg/m^2^ were enrolled. Participants were randomly assigned to one period of oral tadalafil 20 mg once a day and one period of placebo for 6 weeks, separated by an 8-week wash-out period. Placebo and tadalafil tablets were made visually indistinguishable and delivered randomized in two separate boxes for each participant. Both treatment periods ended with a glucose clamp, and measurements of body composition and metabolic markers in blood, subcutaneous and muscular interstitial fluid. The primary aim was to assess difference in whole-body insulin resistance after 6-weeks of treatment, determined after completion of the two study arms, and secondary aims were to study effects of tadalafil on pathophysiology of T2D as well as tolerability of high-dose tadalafil in T2D. Primary analysis was performed in participants with full analysis set (FAS) and safety analysis in all participants who received at least one dose of study medication. This trial is registered with ClinicalTrials.gov (NCT02601989), and EudraCT (2015-000573).

**Findings:**

Between January 22nd, 2016, and January 31st, 2019, 23 participants with T2D were enrolled, of whom 18 were included in the full analysis set. The effect of tadalafil on insulin resistance was neutral compared with placebo. However, tadalafil decreased glycaemia measured as HbA1c (mean difference −2.50 mmol/mol, 95% confidence interval (CI), −4.20; −0.78, p = 0.005), and, further, we observed amelioration of endothelial function and markers of liver steatosis and glycolysis, whereas no statistically significant differences of other clinical phenotyping were shown. Muscle pain, dyspepsia, and headache were more frequent in participants on high-dose tadalafil compared with placebo (p < 0.05) but no difference between treatments appeared for serious adverse events.

**Interpretation:**

High-dose tadalafil does not decrease whole-body insulin resistance, but increases microcirculation, induces positive effects in the liver and in intermediate metabolites, in parallel with an improved metabolic control measured as HbA1c. High-dose tadalafil is moderately well tolerated, warranting larger trials to define the optimal treatment regimen in T2D.

**Funding:**

The 10.13039/501100000265Swedish Research Council, 10.13039/501100008546Swedish Diabetes Foundation, Novo Nordisk Foundation, the Swedish state under the agreement between the Swedish government and the county councils, the ALF-agreement, 10.13039/501100005754Sahlgrenska University Hospital funds, Gothenburg Society of Medicine, 10.13039/100004312Eli Lilly & Company, USA, and Eli Lilly & Company, Sweden AB.

**Research in context**

**Evidence before this study**

We searched PubMed for publications in English using the search terms, "pde5 inhibitor", "type 2 diabetes", and "randomised controlled trial" and identified 2 studies published before Nov 25th, 2022. One study examined effects on urinary albumin-to-creatinine ratio in patients with type 2 diabetes (T2D) and overt nephropathy. The other studied effects on erectile dysfunction (ED) in patients with diabetic neuropathy. Additional articles were identified through stratified searches on PubMed. Studies have demonstrated beneficial effects of phosphodiesterase-5 (PDE-5) inhibitors on insulin resistance and haemoglobin A1c (HbA1c) in men with ED and concomitant T2D. However, studies of PDE-5 inhibitors in men (without ED) and women with T2D are missing.

**Added value of this study**

This is the first study including both men, with no apparent ED, and women with T2D presenting results on a pre-specified metabolic outcome as the primary endpoint. We find no effect of tadalafil on whole-body insulin resistance measured by glucose clamp but demonstrate beneficial metabolic effects through a reduction in HbA1c and a marker of liver steatosis, and improved microcirculation, and peripheral insulin sensitivity. We also report a high frequency of mild to moderate side effects with daily treatment of high-dose tadalafil.

**Implications of all the available evidences**

The study provides support for a beneficial effect on metabolic parameters in T2D through chronic treatment using PDE-5 inhibitors. Large-scale studies should be conducted with HbA1c as the primary endpoint including unselected patients with T2D using longer dose intervals of high-dose tadalafil.

## Introduction

Type 2 diabetes (T2D) is a lethal and debilitating disease with a shorter life span mainly because of a higher incidence of cardiovascular disease.^1^ The risk of myocardial infarction is equally high in individuals with T2D as in non-diabetic individuals who have had their first cardiovascular event.^2^ Endothelial dysfunction is an early pathophysiological derangement for the development of atherosclerosis and T2D.^3^^,^^4^ Blood flow for the supply of hormones and nutrients to muscles is significantly impaired already in pre-diabetic insulin-resistant states due to endothelial dysfunction, as demonstrated by hampered vasodilatory effects of insulin via nitric oxide (NO)-signalling.^5^ In line with this, a large population-based cohort shows that reactive hyperaemia index (RHI), a marker of endothelial function in resistance arteries, is independently associated with metabolic control measured as haemoglobin A1c (HbA1c).^6^ In spite of these reports, there is currently no glucose lowering treatment in clinical use having the main target to reverse endothelial dysfunction in T2D.^7^

Phosphodiesterase-5 (PDE-5) inhibitors constitute a well-established class of drugs targeting endothelial dysfunction through increased NO-signalling, resulting in relaxation of vascular smooth muscles and vasodilation.^8^ This drug class is currently used in the clinic for the treatment of erectile dysfunction, pulmonary hypertension, and lower urinary tract symptoms^9^^,^^10^ but previous registered studies have also suggested cardioprotective effects.^11^^,^^12^ Several preclinical and clinical studies have also demonstrated beneficial metabolic effects of PDE-5 inhibitors for manifestations of the metabolic syndrome.^13^, ^14^, ^15^, ^16^ However, no study with a pre-registered metabolic outcome has examined the effects of PDE-5 inhibition in recently diagnosed well-controlled patients with T2D of both sexes. Tadalafil is a PDE-5 inhibitor with a long half-life^17^ and we hypothesise that an improved endothelial dysfunction and microcirculation induced by high-dose tadalafil once daily would reduce whole-body insulin resistance. Therefore, we set out to evaluate high dose tadalafil in a randomised controlled trial (RCT) for its effect on insulin sensitivity measured by glucose clamp and feasibility as a new treatment option in patients with T2D.

## Methods

### Study design

**Fig. 1:**
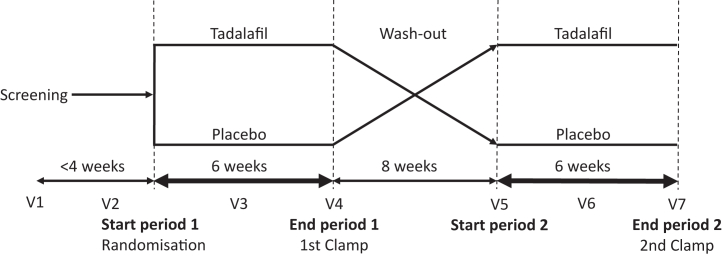
Overview of the study design. After a screening visit, eligible individuals were randomised to either treatment at Visit (V) 2. Treatment period 1 comprises V2, 3 and 4 at 3-weeks interval. Then follows a wash-out period of 8 weeks and a cross-over to treatment period 2 with V5, 6 and 7 during another 6 weeks.

This was a randomised, double-blind, placebo-controlled, single centre, phase II trial using cross-over design in T2D individuals receiving tadalafil 20 mg or placebo once daily for six weeks each with an eight-week wash-out period in between (Fig. 1). All examinations were conducted at the Wallenberg laboratory at Sahlgrenska University Hospital in Gothenburg, Sweden. Each individual was scheduled for seven study visits and was included in the study for 20 weeks (Fig. 1). Before visits two to seven, enrolled individuals fasted overnight and were requested to not take their ordinary medication in the morning nor their study drug and anti-diabetic medication one day prior to the visit. In addition, medication with acetylsalicylic acid (ASA) was suspended one week before visits two to seven. This trial adheres to the CONSORT reporting guidelines and was registered at ClinicalTrials.gov (identifier NCT02601989). The study was approved by the ethical review board in Gothenburg (ID: 291–15) and all the participants provided written consent. The study was registered in the EU Clinical Trials database (EudraCT 2015–000573). All procedures were conducted according to the Declaration of Helsinki.

After the inclusion of four participants, modifications of pre-visit medication withdrawal time were made to improve compliance to study protocol, from three days to one day before visits for the study medication and to apply only for the actual day of the visit for statins and blood pressure medications. Patients with simvastatin were also transferred to rosuvastatin to avoid interactions with tadalafil and the bioimpedance examination was moved from visits three and six to visits four and seven in order to optimise calculations of glucose disposal rate (GDR).

### Participants

Participants were recruited by advertisements in local newspapers and by contacting primary health care centres in Region Västra Götaland in western Sweden (inclusion and exclusion criteria presented in Supplementary Fig. S1). Swedish-speaking females (post-menopause) and males (aged 55–70 years or 40–70 years, respectively), with T2D duration between three months and ten years to ensure a stable metabolic control and few complications, a body mass index (BMI) between 27 and 40 kg/m^2^, and a HbA1c < 60 mmol/mol were recruited. Sex was determined by the third digit in the participants social security number. We asked all male volunteers at screening if they considered themselves to have erectile function of sufficient quality to perform sexual intercourse. All male participants in the study answered “yes” to this question and the result was registered in the study electronic case report form (eCRF). The international Index of Erectile Function (IIEF) for diagnostic evaluation of ED severity was not used during the interviews. Exclusion criteria included medications with known effects on insulin sensitivity or microvascular function, significant microvascular complications or cardiovascular disease, and more than light smoking (detailed in Supplementary Fig. S1).

### Randomisation and masking

The randomisation was conducted by Apoteket Produktion & Laboratorier AB (APL, Stockholm, Sweden). Placebo and tadalafil tablets were made visually indistinguishable to ensure that the study was double-blind. The tablets prepared for oral administration, were delivered in two boxes for each participant, one box containing daily doses of 20 mg tadalafil, and one containing placebo. After inclusion, each participant was given a sequential study identification number and provided with a box corresponding to study number. At the end of period one, the participant returned the box and any remaining tablets. The second box was then delivered at the start of period two. All study personnel, medical staff, and data analysts were blinded to given treatment throughout the study.

### Procedures

#### Anthropometric measurements

Body composition separated into relative fat mass and relative fat-free mass was determined through bioimpedance on a bioelectrical impedance analysis (BIA) (Akern, SMT Medical GmbH, Germany). Body weight was measured in kilogram while height and waist circumference were measured in centimetres. Measurements with bioimpedance were performed at visits two, four, five, and seven. Height was measured at the screening visit while waist circumference and body weight were measured at every visit.

#### Euglycaemic clamp

The infusions of glucose (200 mg/ml) and insulin (120 mU/m^2^/min) was chosen because of the dose response curve for insulin on muscle glucose uptake in insulin-resistant T2D individuals.^18^ Potassium was added to the infusion to avoid hypokalaemia. Steady-state mean plasma glucose was aimed at 5.5 mmol/l at the interval 150–180 min after clamp start by measuring venous plasma glucose concentration bedside with Hemocue® (Ängelholm, Sweden, coefficient of variation (CV) 2.4%) every 5 min throughout the procedure. Mean steady state plasma glucose during tadalafil was 5.5 mmol/l, CV 3.5% and 5.5 mmol/l, CV 4.6% during placebo. If steady-state glucose differed more than 10% between the clamp days in the respective treatment arm, the titration of glucose infusion rate was considered technically insufficient and the individual was excluded from the per-protocol (PP) analysis.

#### Arginine provocation test

Beta-cell response to an intravenous (iv) bolus of arginine was performed after fasting overnight at visits three and six. We administered 5 g bolus of arginine iv at time 0 min and sampling for plasma glucose, serum insulin and C-peptide was performed at baseline and two, three, four, five, six, seven, ten, 25, and 30 min after the injection.^19^

#### Biochemical analyses

Besides safety analyses at the screening visit, we measured HbA1c, fasting glucose, fasting insulin, C-reactive protein (CRP), total cholesterol, high-density lipoprotein (HDL)-cholesterol, low-density lipoprotein (LDL)-cholesterol, fasting triglycerides, free fatty acids (FFA), alanine transaminase (ALT) and aspartate transaminase (AST), alkaline phosphatase (ALP), and bilirubin in venous blood, and albumin creatinine ratio was measured in urine. All samples were analysed using accredited methods at the Clinical Chemistry Laboratory at Sahlgrenska University Hospital in Gothenburg.

#### Blood pressure, forearm blood flow (FBF) and endothelial function measurements

Blood pressure was measured in the left arm after 10 min rest in the supine position (Omron 705 IT, Omron Electronics AB, Kista, Sweden) in the fasting state at every visit and during the glucose clamp at visits four and seven. FBF was measured with venous occlusion plethysmography (D. E. Hokanson, Inc., Washington, United States of America (USA)) before and at 30-min intervals during the clamp at visits four and seven. Endothelial function was measured with the endoPAT 2000 device (Itamar Medical, Caesarea, Israel) at baseline and after three weeks, i.e. at visits two, three, five, and six, for calculation of RHI as previously described.^20^

#### Endothelin-1 (ET-1), interleukin-6 (IL-6) and tumour necrosis factor alpha (TNF-α) measurements

Serum collected after fasting overnight and serum during clamp steady-state were analysed with commercially available assays according to the manufacturer's instructions. We measured serum ET-1 (BioTechne, CV 6.6%), IL-6 (BioTechne, CV 4.9%) and TNF-α (BioTechne, CV 6.7%) (inter-assay) at visits two through seven as well as at clamp steady-state at visits four and seven.

#### Microdialysis measurements

Dialysate concentrations of subcutaneous adipose tissue and muscle insulin levels were collected and measured with a microdialysis technique as previously described.^3^^,^^21^ Dialysate insulin was analysed with an ultrasensitive immunoassay method (Mercodia AB, Uppsala, Sweden). The intra-assay and inter-assay CVs for this method were 5.3% and 6.0%, respectively. Microdialysate samples were collected for measurements of lactate and glycerol concentrations. Glycerol and lactate concentrations in dialysates and plasma were analysed on an ISCUS^flex^ Microdialysis Analyzer, (MDialysis AB, Stockholm, Sweden; CVs 7.6% and 10.4%, respectively).

#### Gene expression analysis of subcutaneous adipose tissue

For the 15 individuals in the PP group, RNA was extracted using RNeasy Lipid Tissue Mini Kit according to the manufacturer's instructions (QIAGEN, Venlo, The Netherlands). Micro-array analysis was conducted on subcutaneous adipose tissue collected at the end of the two treatment periods and performed at the Array and Analysis Facility (Uppsala University). RNA quality was evaluated using the Agilent 2100 Bioanalyzer system (Agilent Technologies Inc, California, USA). Total RNA (250 ng from each sample) was used to generate amplified and biotinylated sense-strand cDNA from the entire expressed genome according to the GeneChip TM WT PLUS Reagent Kit manual Target Preparation for GeneChip TM Whole Transcript (WT) Expression Arrays User Guide (P/N 703174, ThermoFisher Scientific Inc., Life Technologies, California, USA). GeneChip ST Arrays (GeneChip TM Human Gene2.0 ST) were hybridised for 16 h in a 45 °C incubator, rotated at 60 rpm. According to the GeneChip TM Expression Wash, Stain and Scan Manual (PN 702731, Thermo Fisher Scientific Inc.) the arrays were then washed and stained using the GeneChip TM Fluidics Station 450 and finally scanned using the GeneChip TM Scanner 3000 7G. The raw data was normalised in the Transcriptome Analysis Console, version 4.0.2.15, provided by Thermo Fisher, using the Robust Multi-Array Average method (Summarization Method: Gene Level - RMA). Differences in gene expression were tested between tadalafil and placebo treatment, with a 5% false discovery rate and Benjamini-Hochberg correction.

### Calculations

Derived variables were calculated as described below:

*BMI:* Ratio of body weight in kg to the square of height in meters.

*GDR, M-value:* Mean glucose infusion at steady state aiming at plasma glucose 5.5 mmol/l during the last 30 min of the glucose clamp expressed as mg glucose infused per kilogram lean body mass (LBM) per minute.

*M/I value:* The M-value (mg/kgLBM/min) divided by the prevailing insulin concentration (mU/l) at clamp steady state 150–180 min.

*HOMA-IR:* Homeostatic model assessment for insulin resistance index was calculated according to Matthews et al.^22^

### Outcomes

The primary endpoint registered before inclusion of the first participant was difference between treatments in GDR, which equals the M-value in mg/kg LBM/min, determined at steady state (150–180 min) over a 3-h euglycaemic hyperinsulinemic glucose clamp after 6 weeks of 20 mg tadalafil or placebo administered orally once daily. The secondary outcomes registered were; HbA1c and fasting plasma glucose (fP-glucose) levels after 6-weeks, Arginine-induced insulin secretion after 3 weeks, interstitial insulin levels, lactate concentrations, levels of inflammatory markers in blood (specifically ET-1) after 6 weeks, and endothelial function measured with endoPAT (RHI) after 3 weeks. The statistical analysis plan was finalized 2020 01 09, after the start of the study while investigators were still blinded. Secondary objectives were then complemented with additional outcomes on M/I value, fasting serum insulin (fS-Insulin) and HOMA-IR after 6 weeks and arginine-induced c-peptide secretion after 3 weeks. Outcomes on inflammatory markers (CRP, IL-6 and TNF-α), changes in interstitial insulin, lactate and glycerol during the clamp, and alterations in other haemodynamic variables, blood pressure, forearm blood flow and urinary albumin/creatinine ratio after 6 weeks. Circulating markers of liver function and liver steatosis (AST, ALT and ALP), and lipid metabolism were added (total cholesterol, HDL-cholesterol, LDL-cholesterol, FFA and fasting triglycerides), alongside body composition variables (BMI, waist, relative fat mass, relative fat free mass, fat cell diameter) and changes in the subcutaneous adipose tissue gene-expression after 6 weeks (Supplementary material, Statistical Analysis Plan, section 1.1).

### Statistical analysis

Due to invasive and time-consuming techniques used for the assessment of several of the study outcomes, a cross-over design was chosen to limit the number of participants for recruitment. A power calculation was performed with GDR in skeletal muscle during glucose clamp as main outcome parameter with a power of 90% and a significance level of 0.05. Based on previous clamp studies in our laboratory, a standard deviation (SD) of 1.50 mg/kgLBM/min was found for differences in patients with T2D and a mean difference of 1 mg/kgLBM/min between tadalafil and placebo treatments was anticipated. With Fisher's permutations test for paired observations and these assumptions, 20 patients with T2D in total were calculated as sufficient to find statistical differences between the two study arms. We assumed a drop-out rate of ca 20% due to side effects and therefore we aimed to randomise 25 patients with T2D.

Categorical variables are presented as n (%) and continuous variables as mean (SD or 95% confidence interval (CI) as indicated). Treatment induced changes for all outcomes are presented as mean (CI). Period-adjusted p-values were calculated by analysing change from period one to period two (between treatment) using Fisher's non-parametric permutation test. For comparison over time, a linear non-parametric permutation test for paired observations was used for continuous variables. For any given variable, the n value can sometimes vary between the two treatment arms as well as comparisons between treatments due to technical complications. The range of n between these analyses is therefore presented. Missing data were not imputed for any analysis.

No subgroup analysis was made and all p-values presented are period-adjusted p-values comparing tadalafil treatment with placebo, unless otherwise stated. Further, no interim analysis was performed and no specific stopping guidelines were formed as tadalafil has been in clinical practice for many years and was administered within the therapeutic range. All statistical analyses were performed using SAS® v9.2 (Cary, NC, USA).

### Role of the funding source

The funder of the study had no role in study design, data collection, data analysis, data interpretation, or writing of the report. E.F., V.R.R.S., L.S., M. B-T., L-M.G; and P-A.J have accessed and verified the underlying data. P-A.J. was responsible for the decision to submit the manuscript and is the guarantor of this work.

## Results

### Participants

**Fig. 2:**
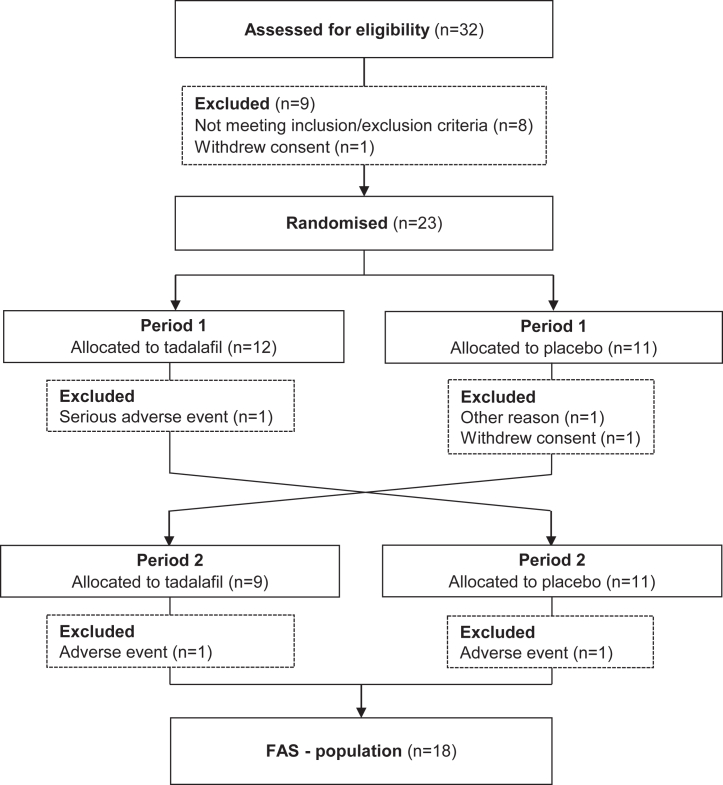
Flowchart of the study. A phase II, double-blind, randomised, placebo-controlled cross-over, single centre, 6-week trial. Full analysis set (FAS).

Study participation is presented in Fig. 2. Out of 32 individuals who attended screening, nine did not meet the pre-specified inclusion criteria and 23 individuals were included in the study and the safety data analysis. One participant initiated the study with active treatment, experienced pyelonephritis early on leading to a brief hospitalisation for iv antibiotic treatment, resulting in a serious adverse event (SAE) and withdrawal from the study prior to the first glucose clamp. Pyelonephritis is not a known side-effect of tadalafil. Additionally, four participants did not complete the study, excluding them from the full analysis set (FAS) (n = 18). One participant reported muscular back pain and burning feet while on active treatment in period one, which was relieved during wash-out, but terminated the study in period two due to a persistent cold while on placebo. Another participant terminated due to muscular back pain after three days of active treatment in period two. Another participant withdrew consent because of a serious illness in the family, and finally, one participant was excluded due to significant changes in lifestyle and consequent hyperglycaemia, while in the placebo arm. Medical history and concomitant medication of the FAS population are shown in Supplementary Tables S1 and S2. Fifteen participants were included in the per protocol (PP) analysis, as one participant had been incorrectly included due to a misunderstanding of medical history, one participant could not achieve stable steady state glucose of 5.5 mmol/l during one of the glucose clamps, and one participant presented <80% exposure to active treatment. The first participant was enrolled on January 22nd, 2016, and the last visit for a participant in the study was on January 31st, 2019. The trial was closed at the time of expiration of the study medications. Participant characteristics for the FAS population are presented in Table 1. Two-thirds of the participants were male and one-third were female. The mean (SD) age was 63 (6) years, BMI 31.4 (3.7) kg/m^2^, diabetes duration 4 (3) years, fasting glucose 6.8 (1.3) mmol/l and HbA1c of 46 (6) mmol/mol.

**Table 1:** Baseline characteristics of the full analysis set population, n = 18.

	Participants
Male, n (%)	12 (67)
Female, n (%)	6 (33)
Age, years	63.3 (5.9)
BMI, kg/m^2^	31.4 (3.7)
Waist, cm	109 (9)
Systolic blood pressure, mmHg	145 (14)
Diastolic blood pressure, mmHg	88 (7)
Diabetes duration, years	4 (3)
Never smoked, n (%)	8 (44)
Former smoker, n (%)	10 (56)
P-Glucose, mmol/l	6.8 (1.3)
B-HbA1c, mmol/mol	46 (6)
S-Cholesterol, mmol/l^a^	5.0 (1.1)
S-LDL, mmol/l^b^	3.4 (1.0)
S-HDL, mmol/l^b^	1.2 (0.3)
S-Triglycerides, mmol/l^b^	1.91 (0.67)
U-Albumin, mg/l	37.2 (59.2)
S-Creatinine, μmol/l^b^	79 (17)
S-ALT, μkat/l^a^	0.61 (0.21)
S-AST, μkat/l^a^	0.46 (0.11)

Note: For categorical variables n (%) is presented.

For continuous variables mean and standard deviation (SD).

Abbreviations: BMI = Body mass index; P-Glucose = Plasma glucose; B-HbA1c = Blood haemoglobin A1c; S-Cholesterol = Serum cholesterol; S-LDL = Serum low-density lipoprotein; S-HDL = Serum high-density lipoprotein; S-Triglycerides = Serum triglycerides; U-Albumin = Urine albumin; S-Creatinine = Serum creatinine; S-ALT = Serum alanine aminotransferase; S-AST = Serum aspartate aminotransferase.

a Measured at visit 2 in 2 individuals due to technical failure at screening.

b Measured at visit 2 in 3 individuals due to technical failure at screening.

### Insulin resistance and metabolic control

**Fig. 3:**
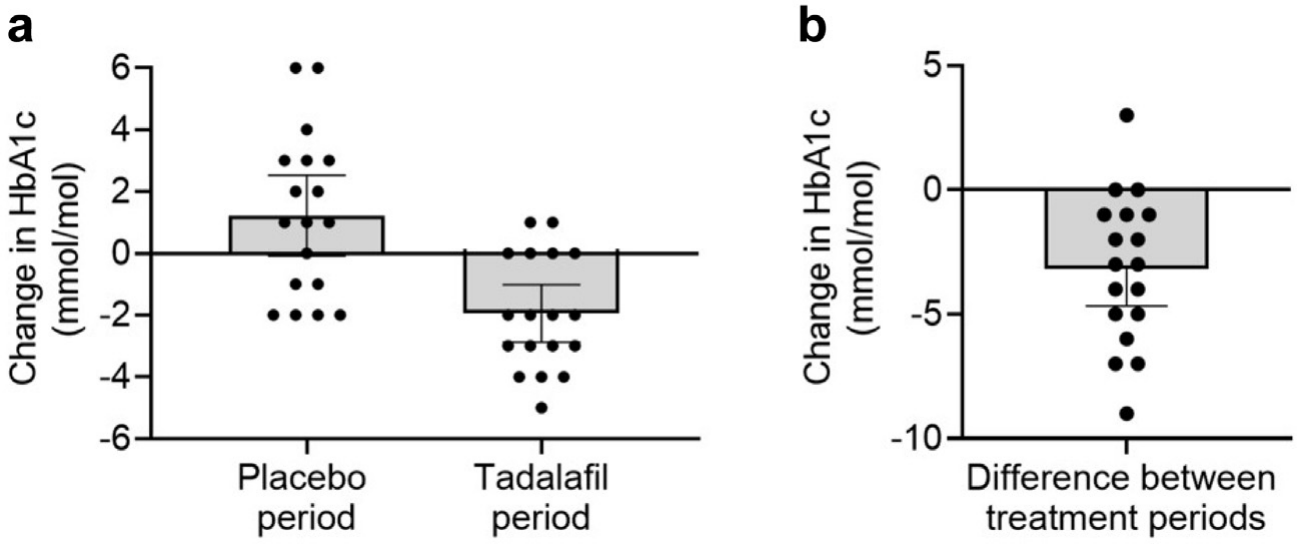
Tadalafil treatment (20 mg orally once daily for 6 weeks) significantly reduces Haemoglobin A1c (HbA1c) compared to placebo treatment. (a) Change in HbA1c over respective treatment period (n = 18, full analysis set (FAS) population). (b) Difference in HbA1c-change between periods, comparing placebo and tadalafil treatment for every individual (n = 18, FAS population). Data are presented as mean (95% confidence interval (CI)).

Tadalafil did not present significant effects on the primary outcome defined as improvement in GDR during an euglycaemic hyperinsulinemic glucose clamp compared to placebo (Table 2). The M-value in the tadalafil and placebo group was similar (mean difference 0.467 mg/kgLBM/min, 95% CI, −0.450; 1.43, p = 0.39). However, there was a significant period-adjusted reduction of HbA1c (mean difference −2.50 mmol/mol, 95% CI, −4.20; −0.78, p = 0.0051) (Table 2). Individual changes in HbA1c for all participants in the FAS population are demonstrated for the tadalafil and placebo periods, as well as the change between periods in Fig. 3a and b. End of period HbA1c in the tadalafil and placebo groups were 44.4, 95% CI (41.7; 47.2) and 46.9, 95% CI (43.3; 50.5) mmol/mol. Tadalafil did not affect M/I-value (p = 0.19), fasting glucose (p = 0.24), fasting insulin (p = 0.38) or HOMA-IR (p = 0.15). Tadalafil did not have any significant effect on the primary endpoint in the PP population, however, the significant decrease of HbA1c was persistent in the PP population (p = 0.031).

**Table 2:** Effect of tadalafil on primary endpoint, markers of insulin resistance and metabolic control in the full analysis set population, n = 18.

Variable	Placebo	Tadalafil 20 mg	Change from Placebo to Tadalafil 20 mg	p-value	Period-adjusted p-value
M-value (mg/kgLBM/min):	8.71 (7.32; 10.12)	9.18 (7.69; 10.67)	0.467 (−0.450; 1.431)	0.32	0.39
M/I value (100mgl/kg/min/mU)	3.50 (2.82; 4.18)	3.85 (2.95; 4.74)	0.342 (−0.147; 0.841)	0.16	0.19
B-HbA1c (mmol/mol)	46.9 (43.3; 50.5)	44.4 (41.7; 47.2)	−2.50 (−4.20; −0.78)	**0.0087**	**0.0051**
fP-Glucose (mmol/l)	7.66 (6.98; 8.34)	7.37 (6.73; 8.06)	−0.289 (−0.740; 0.157)	0.19	0.24
fS-Insulin (mU/l)	17.2 (14.1; 20.2)	16.5 (13.2; 19.8)^1^	−0.876 (−3.311; 1.612)^1^	0.46	0.38
HOMA-IR Index	5.83 (4.69; 6.97)	5.26 (4.07; 6.49)^1^	−0.545 (−1.317; 0.262)^1^	0.17	0.15

Note: M-value was calculated per kg lean body mass.

M/I value was calculated per kg LBM (100mgl/kg/min/mU) divided by the prevailing insulin concentration at clamp steady state.

For continuous variables, mean and 95% confidence interval (CI) are presented.

Period-adjusted p-value was calculated by standard cross-over analysis method using Fisher's non-parametric permutation test. Bold values denote statistical significance at the p < 0.05 level.

Abbreviations: LBM = Lean body mass; B-HbA1c = Blood haemoglobin A1c; fP-Glucose = Fasting plasma glucose; fS-Insulin = Fasting serum insulin; HOMA-IR = Homeostatic model assessment of insulin resistance.

^1^n = 17.

### Endothelial function, blood pressure, FBF and urine albumin excretion

Tadalafil significantly improved the endothelial function measured as RHI (mean 0.306, 95% CI, 0.083; 0.525, p = 0.0064) (Table 3). There were no effects of tadalafil on FBF (p = 0.75), systolic or diastolic blood pressure (p = 0.58 and 0.23, respectively) or Urine albumin (U-albumin)/creatinine ratio (p = 0.22) compared with placebo. Post hoc analysis revealed a correlation between RHI during treatment and end of treatment HbA1c (r = −0.39, p = 0.020).

**Table 3:** Effects of tadalafil on clinical and metabolic characteristics in the full analysis set population, n = 18.

Variable	Placebo (n = 18)	Tadalafil 20 mg (n = 18)	Change from Placebo to Tadalafil 20 mg	p-value	Period-adjusted p-value
**Body composition**					
Weight (kg)	94.0 (86.9; 101.3)	94.3 (87.1; 101.4)	0.22 (−0.35; 0.79)	0.41	0.52
Waist (cm)	108.9 (104.5; 113.4)	109.0 (104.4; 113.6)	0.06 (−1.62; 1.72)	0.97	0.86
BMI, kg/m^2^	31.3 (29.4; 33.3)	31.5 (29.5; 33.4)^1^	0.06 (−0.13; 0.26)^1^	0.53	0.55
Body fat (%)	36.0 (33.3; 38.7)	35.1 (32.1; 38.1)^1^	−1.01 (−2.20; 0.15)^1^	0.093	0.062
Lean body mass (%)	64.8 (61.9; 67.7)	65.4 (62.0; 68.7)^1^	0.66 (−1.36; 2.64)^1^	0.50	0.62
Fat cell diameter (μm)	112.9 (107.5; 118.2)^6^	111.4 (108.0; 114.8)^6^	−1.50 (−5.75; 2.67)^6^	0.46	0.44
**Vascular function**					
FBF (ml/100 g/min)	4.13 (3.10; 5.18)^2^	4.00 (3.47; 4.54)^1^	−0.07 (−0.97; 0.84)^2^	0.86	0.75
SBP (mmHg)	137.9 (130.6; 145.1)	139.3 (131.7; 147.0)	1.44 (−3.86; 6.75)	0.63	0.58
DBP (mmHg)	86.3 (82.1; 90.5)	83.8 (80.0; 87.7)	−2.50 (−6.62; 1.67)	0.20	0.23
RHI	2.01 (1.77; 2.24)	2.31 (2.04; 2.59)	0.306 (0.083; 0.525)	**0.0090**	**0.0064**
U-Albumin/U-Creatinine ratio	5.48 (0.46; 11.49)	3.12 (0.58; 6.38)	−2.36 (−5.61; 0.06)	0.073	0.22
**Liver function**					
S-ALP (μkat/l)	1.13 (1.01; 1.25)	1.07 (0.97; 1.18)	−0.058 (−0.118; 0.000)	0.053	0.064
S-AST (μkat/l)	0.394 (0.337; 0.451)^1^	0.411 (0.346; 0.479)^1^	0.017 (−0.017; 0.051)^1^	0.35	0.43
S-ALT (μkat/l)	0.575 (0.445; 0.709)	0.495 (0.380; 0.612)	−0.080 (−0.127; −0.034)	**0.0019**	**0.0013**
**Lipids**					
S-Cholesterol (mmol/l)	4.82 (4.20; 5.43)	4.83 (4.24; 5.41)	0.082 (−0.180; 0.317)	0.57	0.58
S-HDL (mmol/l)	1.18 (1.03; 1.33)^1^	1.18 (1.03; 1.34)^1^	0.001 (−0.044; 0.047)^2^	0.95	0.87
S-LDL (mmol/l)	3.25 (2.70; 3.79)	3.28 (2.73; 3.82)	0.003 (−0.167; 0.220)	0.77	0.79
S-Triglycerides (mmol/l)	1.84 (1.46; 2.23)	1.65 (1.83; 1.97)^1^	−0.158 (−0.474; 0.117)^1^	0.39	0.40
FFA (mmol/l)	0.538 (0.478; 0.596)	0.511 (0.420; 0.601)	−0.027 (−0.086; 0.032)	0.35	0.37
**Beta-cell function**					
iAUC S-Insulin, 0–30 min (mU/l)	435.4 (334.4; 537.4)	486.1 (367.6; 611.0)^1^	39.1 (−52.1; 136.4)^1^	0.38	0.42
iAUC C-Peptide, 0–30 min (nmol/l)	9.58 (7.86; 11.29)	10.2 (8.5; 11.9)^1^	0.55 (−0.30; 1.41)^1^	0.53	0.54
**Metabolic measurements during glucose clamp**					
S-Insulin, basal to 180 min (mU/l)	249.2. (291.3; 277.5)	255.0 (232.2; 276.7)	5.83 (−12.50; 24.37)	0.44	0.50
D-Insulin sc, basal to 180 min (mU/l)	5.2 (4.4; 6.1)^5^	4.4 (3.5; 5.3)^7^	−0.98 (−1.60; −0.37)^7^	0.080	0.12
D-Insulin muscle, basal to 180 min (mU/l)	3.0 (2.1; 4.0)^6^	2.8 (2.0; 3.6)^7^	−0.24 (−2.10; 1.57)^8^	0.81	0.70
P-Glycerol, basal to 180 min (μmol/l)	−41.1 (−51.0; −31.3)	−39.5 (−47.8; −31.5)	−1.61 (−6.75; 10.50)	0.63	0.52
I-Glycerol, basal to 180 min (μmol/l)	−109.2 (−141.8; −76.8)^3^	−109.4 (−135.3; −84.1)^2^	−0.24 (−32.5; 32.1)^4^	0.87	0.65
P-Lactate, basal to 180 min (μmol/l)	201.1 (−1.7; 401.3)	399.8 (−250.1; 548.5)	198.7 (53.1; 358.3)	**0.0078**	**0.0078**
I-Lactate, basal to 180 min (μmol/l)	918 (496; 1338)^1^	1014 (734; 1295)^2^	93.3 (367.3; 569.3)^2^	0.67	0.79
P-Glycerol, 180 min (μmol/l)	14 (10.5; 17.5)	10.9 (8.4; 13.5)	−3.1 (−5.2; −0.9)	**0.012**	0.069
P-Lactate, basal (μmol/l)	767 (613; 932)	638 (562; 718)	−128.8 (−262.6; 0.7)	0.071	**0.025**
**Inflammation**					
S-CRP (mg/l)	3.7 (1.8; 5.7)	5.6 (2.0; 10.6)	1.86 (−1.59; 6.31)	0.52	0.52
S-Endothelin-1 (pg/ml)	1.3 (0.9; 1.6)	1.2 (0.9; 1.5)	−0.08 (−0.21; 0.05)	0.29	0.27
S-TNF-α (pg/ml)	1.2 (1.1; 1.4)	1.2 (1.0; 1.3)	−0.05 (−0.17; 0.06)	0.37	0.27
S-IL-6 (pg/ml)	3.6 (2.3; 5.0)	3.6 (2.3; 5.4)	0.11 (−1.31; 1.57)	0.81	0.82

Note: For continuous variables, Mean and 95% confidence interval (CI) are presented.

Period-adjusted p-value was calculated by standard cross-over analysis method using Fisher's non-parametric permutation test. Bold values denote statistical significance at the p < 0.05 level.

Basal is an average of measurements at time −60, −30, −15 and 0 min.

Data missing due to technical failures, ^1^n = 17, ^2^n = 16, ^3^n = 15, ^4^n = 14, ^5^n = 13, ^6^n = 12, ^7^n = 11, ^8^n = 9.

Abbreviations: BMI = Body mass index; FBF = Forearm blood flow; SBP = Systolic blood pressure; DBP = Diastolic blood pressure; RHI = Reactive hyperaemia index; U-Albumin = Urine albumin; U-Creatinine = Urine creatinine; S-ALP = Serum alkaline phosphatase; S-ALT = Serum alanine aminotransferase; S-AST = Serum aspartate aminotransferase; S-Cholesterol = Serum cholesterol; S-LDL = Serum low-density lipoprotein; S-HDL =Serum high-density lipoprotein; S-Triglycerides = Serum triglycerides; FFA = Free fatty acids; iAUC = incremental area under the curve; S-Insulin = Serum insulin; D-Insulin sc = Dialysate insulin subcutaneous; D-Insulin muscle = Dialysate insulin muscle; P-Glycerol = Plasma glycerol; I-Glycerol = Interstitial glycerol; P-Lactate = Plasma lactate; I-Lactate = Interstitial lactate; S-CRP = Serum C-reactive protein; S-Endothelin-1 = Serum endothelin-1; S-TNF-α = Serum tumour necrosis factor alpha; S-IL-6 = Serum interleukin-6.

### Body composition and microarray of subcutaneous adipose tissue

There was no significant effect of tadalafil on body weight (p = 0.52), waist circumference (p = 0.86), BMI (p = 0.55), or lean body mass (p = 0.62) (Table 3). However, the influence of tadalafil treatment on reduced body fat percentage after six weeks was close to the significance threshold (p = 0.062). Further, microarray analyses (false discovery rate 5%) in the subcutaneous adipose tissue did not reveal any significant alterations in gene expression between tadalafil and placebo treatment. Several genes specifically known to regulate glucose and lipid metabolism, thermogenesis and adipose tissue differentiation were also examined individually, but did not differ between study arms (data not shown).

### Liver function, blood lipids, beta-cell function and inflammatory markers

The fatty liver marker ALT was significantly reduced by tadalafil treatment compared with placebo, (mean 0.080 μkat/l, 95% CI, −0.127; −0.034, p = 0.0019) (Table 3). No statistically significant effect of tadalafil compared with placebo was seen for other markers of liver function (AST, p = 0.064 and ALP, p = 0.064). There were no statistically significant effects of tadalafil compared with placebo treatment on total cholesterol (p = 0.58), HDL-cholesterol (p = 0.87), LDL-cholesterol (p = 0.79), triglycerides (p = 0.40) or FFA (p = 0.37). Three weeks of tadalafil treatment did not statistically significantly improve markers of arginine-stimulated insulin secretion (Table 3), (incremental Area Under Curve (IAUC) S-Insulin 0–30 min, p = 0.42; IAUC serum insulin (S-Insulin)/plasma glucose (P-Glucose) 0–10 min, p = 0.074), and C-peptide secretion (IAUC serum C-peptide (S–C-Peptide) 0–30 min, p = 0.54; IAUC S–C-Peptide/P-Glucose 0–10 min, p = 0.20). Finally, tadalafil did not reduce the inflammatory markers CRP (p = 0.52), ET-1 (p = 0.27), TNF-α (p = 0.27) nor IL-6 (p = 0.82).

### Metabolic alterations during glucose clamp

Tadalafil did not affect circulating or muscular dialysate insulin levels at clamp steady state compared with placebo (p = 0.50 and p = 0.77, respectively), and did not change dialysate insulin concentration during microdialysis of subcutaneous adipose tissue (p = 0.080) (Table 3). Further, no change was seen in interstitial lactate dynamics during the glucose clamp, however, tadalafil did result in an enhanced plasma lactate response during the glucose clamp (mean 198.7 μmol/l, 95% CI, 53.1; 358.3, p = 0.007) and decreased fasting plasma lactate concentration (mean −128.8 μmol/l, 95% CI, −262.6; 0.7, p = 0.025). After glucose clamp, the tadalafil group had lower plasma glycerol levels before (p = 0.012), but not after, adjustment for treatment period (mean −3.1 μmol/l, 95% CI, −5.2; −0.9, p = 0.069) (Table 3).

### Adverse events

The adverse event categories, gastrointestinal disorders, general disorders and administration site conditions, musculoskeletal and connective tissue disorders, and nervous system disorders, were more frequent during treatment with tadalafil compared with placebo (Table 4). The most frequently reported symptoms within these categories were headache (48%), dyspepsia (33%), gastroesophageal reflux (24%), arthralgia (24%), back pain (24%) and pyrexia (feverish sensation but normal body temperature, 14%) (Supplementary Table S3).

**Table 4:** Type of adverse events registered in the safety population, n = 23.

	Participants with events on placebo (n = 22) n (%)	Participants with events on tadalafil (n = 21) n (%)	*p*-value
Any event	14 (63.6%)	19 (90.5%)	0.069
Cardiac disorders	1 (4.5%)	0 (0%)	>0.99
Eye disorders	0 (0%)	3 (14.3%)	0.11
Gastrointestinal disorders	6 (27.3%)	15 (71.4%)	**0.0059**
General disorders and administration site conditions	1 (4.5%)	6 (28.6%)	**0.046**
Immune system disorders	1 (4.5%)	0 (0%)	>0.99
Infections and infestations	7 (31.8%)	6 (28.6%)	0.99
Injury, poisoning and procedural complications	3 (13.6%)	1 (4.8%)	0.61
Metabolism and nutrition disorders	1 (4.5%)	0 (0%)	>0.99
Musculoskeletal and connective tissue disorders	8 (36.4%)	15 (71.4%)	**0.033**
Nervous system disorders	5 (22.7%)	12 (57.1%)	**0.031**
Psychiatric disorders	0 (0%)	2 (9.5%)	0.23
Respiratory, thoracic and mediastinal disorders	1 (4.5%)	5 (23.8%)	0.095
Skin and subcutaneous tissue disorders	1 (4.5%)	2 (9.5%)	0.61
Surgical and medical procedures	2 (9.1%)	1 (4.8%)	>0.99
Vascular disorders	0 (0%)	2 (9.5%)	0.23

Statistical differences were calculated with Fisher's exact test. Bold values denote statistical significance at the p < 0.05 level.

## Discussion

We performed a double-blind RCT in patients with T2D and assessed whether tadalafil is better than placebo to reverse insulin resistance, and feasible as an antidiabetic agent in T2D. Tadalafil did not significantly improve insulin resistance expressed as GDR during glucose clamp. However, metabolic control measured as HbA1c was alleviated in parallel with metabolic variables. Endothelial function was enhanced by tadalafil compared to placebo and improvement of RHI was associated with decrease of HbA1c in patients with T2D. Furthermore, the liver steatosis marker ALT was attenuated by tadalafil compared to placebo. Tolerability of high-dose tadalafil treatment was moderate among study participants; two participants withdrew consent because of back pain and burning sensations in the lower extremities. Furthermore, reversible side effects such as headache, muscle pain and dyspepsia were quite frequent and had to be reversed by temporary on-demand medication.

Our study is the first to evaluate whether chronic treatment with a PDE-5 inhibitor improves insulin sensitivity in a predefined analysis plan in men and women with T2D. Defining the M-value from the euglycaemic glucose clamp as the primary endpoint, we found no effect in patients with T2D. In contrast, an RCT including obese individuals with prediabetes reported an increase in insulin sensitivity index assessed with a hyperglycaemic glucose clamp, during treatment with sildenafil 25 mg for three times daily compared to placebo in a parallel design study over three months.^15^ Differences in type and dosages of PDE-5 inhibitor, length of stop of study medication use before the clamp, study population and methods to assess insulin sensitivity may explain the difference in results.

We found that tadalafil reduced HbA1c levels in this study on men and women with T2D. Recently, Lee et al. performed a placebo-controlled, parallel group, six-months RCT of low-dose tadalafil in men with erectile dysfunction and T2D, to evaluate metabolic effects. The authors reported significantly lower HbA1c levels during active treatment, detailing a 0.14% (1–2 mmol/mol) decrease in HbA1c compared to baseline during tadalafil treatment whereas HbA1c increased in the placebo group.^23^ Other studies have also reported that PDE-5 inhibitors can lower HbA1c levels in patients with T2D in studies designed for non-metabolic outcomes. An RCT on the kidney protective effect of a novel long-acting PDE-5 inhibitor or placebo for 12 weeks observed modest positive effects on HbA1c.^24^ In contrast, an RCT in 60 patients with T2D who were randomised to sildenafil 100 mg/day or placebo for three months observed decreased HbA1c levels by 0.5–0.7% in both groups.^25^ Although a short treatment period, we also report a reduction in HbA1c compared to placebo in a majority of both male and female patients with T2D.

Tadalafil improved markers of endothelial function. This has been previously reported in human studies where acute administration of tadalafil showed positive effects on capillary recruitment in muscle and forearm glucose uptake in patients with T2D.^26^ Indeed, in this study, change in endothelial function, registered as RHI, correlated with improved metabolic control in the study participants. We did not find any changes in other haemodynamic variables, and while other studies have reported improvements in albuminuria induced by PDE-5 inhibition, this was not found in this study.^15^^,^^24^^,^^27^ Tadalafil acts on the microcirculation, and the observed difference in RHI between tadalafil and placebo is well explained by increased NO-signalling following drug administration. Furthermore, our participants had a well-controlled T2D, and this is likely the reason we did not see changes in albuminuria.

Positive effects on ALT levels induced by tadalafil in this study are in agreement with the recent publication by Lee et al.^23^ In line with this trial, the observation was not accompanied by any statistically significant effects on reduction of body fat, waist circumference or markers of lipid metabolism. In contrast, a previous study with sildenafil 100 mg/day for three months in patients with T2D resulted in decreased waist circumference, attenuation of ectopic lipid deposition shown as visceral adipose tissue and epicardial adipose tissue measured with magnetic resonance imaging.^25^ Another recent study on PDE-5 inhibition in mice presented a reduction of liver steatosis markers, serum triglycerides and macrophages in the liver and the suggested explanations for this were increased lipolysis and increased beta-oxidation in liver cells.^28^ Our extensive phenotyping including gene array analysis did not indicate any distinct effect on white adipose tissue as a mediator of ALT-reduction by tadalafil in patients with T2D. Therefore, this observation deserves further study.

It may appear contradictory that we did not find any significant effect of tadalafil on insulin resistance assessed by glucose clamp in this study, when observing a decrease in HbA1c. However, insulin resistance is a complex pathophysiological process, and several variables can be used to quantify the different aspects. While the M-value mainly measures muscular insulin resistance, the HOMA-IR mainly reflects hepatic insulin resistance.^29^ In this study, the HOMA-IR indicated a beneficial direction of effect by tadalafil on IR, although not statistically significant. Recently, Lee et al. noticed that tadalafil suppressed fasting plasma glucose in patients with T2D.^23^ It is possible that studies on individuals with higher HOMA-IR and HbA1c could find more pronounced effect on fasting glucose levels and insulin resistance measured by HOMA-IR. If tadalafil exerts its beneficial effects on HbA1c through effects on the liver, the beta-cells or through other mechanisms deserves to be examined in designated studies. We suggest that future RCT should include infusion of tritiated glucose or stable isotopes to evaluate effects of tadalafil on hepatic glucose production and insulin resistance.

During the glucose clamp, treatment with tadalafil improved plasma lactate dynamics, a variable reflective of increased glycolysis and glucose uptake in the periphery. Plasma lactate in the basal state was also more suppressed in tadalafil compared to placebo-treated patients in line with a trend of less percent body fat after tadalafil treatment. Insulin-induced suppression of lipolysis, measured as plasma glycerol after clamp, was significantly improved in the unadjusted analysis, possibly indicating an effect in the adipose tissue. Indeed, there is a role of adipose tissue, besides skeletal muscle, for glucose uptake and lactate metabolism.^30^ However, *in situ* measurements of lactate in adipose tissue showed no difference in results between the two study arms. Unfortunately, we had no measurements of adipose tissue blood flow, preventing proper evaluation of subcutaneous lactate and glycerol release. It is possible that increased plasma lactate and decreased plasma glycerol levels during the glucose clamp indicate an increase in insulin sensitivity induced by tadalafil in patients with T2D.

We also investigated the effects of tadalafil in the pancreas and its modulation of circulating markers of relevance in the pathophysiology of T2D. No clear effect of tadalafil on markers of beta-cell function were observed, which is in contrast to a previous study on tadalafil treatment in patients with the metabolic syndrome.^14^ We observed no decrease of circulating inflammatory markers by tadalafil treatment. Conversely, a meta-analysis on PDE-5 inhibition in T2D has previously shown that sildenafil treatment suppressed circulating IL-6 concentrations.^13^ Taken together, we could not replicate many of the previous results reported on PDE-5 inhibition in patients with T2D, possibly due to differences in study design.

Tolerability of chronic high-dose tadalafil treatment was moderate in our patient cohort with ca 10% drop-out from the study due to adverse events. Dyspepsia, headache and myalgia of moderate to high intensity required temporary medication for reversal of the symptoms in ca 50% of the participants. Typical clinical signs of high dose tadalafil treatment also included transient hyperaemia with flush and a warm sensation in the upper body as well as nasal congestion. Previous investigations on high-dose tadalafil treatment in obese or patients with T2D have reported similar symptoms but milder side effects compared to this trial.^31^ In the near future, high-dose tadalafil may be tested for its efficacy on metabolic control, and side effects evaluated at lower doses or longer intervals of treatment.

We have some limitations to consider. This is a small trial performed over a relatively short period of time and therefore we had limited power to detect modest effects of tadalafil. Moreover, according to our power analysis, we did not achieve enough participants in the FAS population for our primary endpoint. Nonetheless, HbA1c was significantly reduced after tadalafil compared with placebo and in some individuals to a remarkable degree. Further, seven participants had their medication changed from angiotensin converting-enzyme inhibitor and angiotensin II receptor blocker (ACEi/ARB) to Calcium Channel Blockers (CCB) or diuretics five to eleven days before starting the respective treatment arm at visit two and visit five. Changed medications were checked off with the participants’ ordinary general physician and performed under careful monitoring of blood pressure. No apparent difference in the FAS population appeared as to the effect of tadalafil on GDR during glucose clamp and the effect of tadalafil on HbA1c compared with placebo, whether blood pressure medication was changed before the start of the respective treatment arms, or not. However, we cannot exclude that other secondary endpoints comparing the effect of tadalafil and placebo were affected by this medication change. The small sample size of the study, and multiple secondary outcomes increase the risk of false positive associations. This motivates larger confirmatory studies with HbA1c as the primary outcome. Monitoring of interstitial glycerol and lactate concentration in subcutaneous adipose tissue were hampered by technical problems with several of the microdialysis catheters and hence we had low power for detection of differences between study arms for these variables. Adipose tissue blood flow was not obtained because of problems to get Xenon in Europe and that was a limitation for our interpretation of tadalafil effects in adipose tissue. Furthermore, dialysate insulin as an estimate of tissue levels had high variability and the compound inulin, important for more precise interstitial measurements, was no longer commercially available.^3^^,^^21^ Energy balance could not be assessed because we did not register dietary intake, monitor physical activity or include the doubly labelled water method. All participants were told to keep their lifestyle after entering the study and, indeed, participants were weight stable during the treatment arms with tadalafil or placebo. Since this is a study with a cross-over design, carry-over effects must be considered. However, the elimination half-life of tadalafil is 17.5 h and the wash-out period was eight weeks and the statistics presented were also period-adjusted. Taken together, these steps should minimise this risk of any carry-over effects. This trial was conducted on a Caucasian study population and it is possible that other effects induced by tadalafil may be seen in participants of other ethnicities. Our study included both men and women, although the small sample size did not allow stratified analysis to investigate any potential influence of sex on the treatment. We did not include participants with erectile dysfunction to improve the masking of active treatment. However, it is well known that both erectile dysfunction and low testosterone levels are common in obesity and T2D.^32^ Therefore, it is possible that studies specifically recruiting individuals with several of these comorbidities may find additional benefits from tadalafil treatment also metabolically. Finally, although the study was double-blinded, it is possible that the investigator and participant could have been biased by recognising symptoms typical of tadalafil treatment. We asked the participants not to change their lifestyle during the study. However, we cannot exclude that participants on tadalafil treatment could have sexual intercourse more frequently compared with the placebo treatment period with possible impact on metabolic outcomes.

Thus, although high dose tadalafil did not increase insulin sensitivity during glucose clamp in patients with T2D, positive metabolic effects were induced in the liver and endothelium and in intermediate metabolites, in parallel with reduced glycaemia measured as lower HbA1c levels. High dose tadalafil was moderately tolerated and 50% of the patients with T2D developed headache, dyspepsia and back pain that required temporary medication. Larger long-term studies are warranted to address whether high dose tadalafil sustains its positive metabolic effects and acceptable side effects in patients with T2D if administered at longer intervals, e.g., three times per week.

## Contributors

P-A.J conceived the MAKROTAD study; E.F., V.R.R.S., L.S., M. B-T., and P-A.J. collected and analysed clinical data. M.S. contributed to subcutaneous microdialysis and L-M.G. contributed to measurements of microcirculation. E.F., V.R.R.S. and P-A.J. interpreted the results and wrote the manuscript with input and comments from the others and all authors approved the final version of the manuscript and agree to be accountable for all aspects of the work.

## Data sharing statement

Full copies of the signed study protocol and statistical analysis plan are found as supplementary material. Normalized data from the subcutaneous adipose tissue micro-array run are deposited at https://www.ebi.ac.uk/biostudies/arrayexpress (accession E-MTAB-12891). Data collected for the study will be made available to scientific peers through contact with the corresponding author, within certain limitations. For sharing of data within a scientific collaboration, please e-mail any proposal to the corresponding author. Data will only be shared in accordance with legal frameworks, and when the integrity of the individual study participant can be guaranteed. This will be decided by the corresponding author on a case-by-case basis.

## Declaration of interests

All authors declare no competing interests. Professor Gan holds an employment of Ribocure Pharmaceuticals AB, but has no conflict of interest in relationship to this study.

## Appendix A Supplementary data

Supplementary material

Translated abstract
