# Supplementary material for: Feasibility of high-dose tadalafil and effects on insulin resistance in well-controlled patients with type 2 diabetes (MAKROTAD): a single-centre, double-blind, randomised, placebo-controlled, cross-over phase 2 trial
Source: eClinicalMedicine. 2023 May 4;59:101985. doi: 10.1016/j.eclinm.2023.101985 (PMC10225663; doi:10.1016/j.eclinm.2023.101985)
Supplement: Supplementary material [file mmc1.pdf]

Supplementary material

Supplementary Figure 1

To be eligible for the study, participants were required to fulfil the following inclusion criteria:
1. Type 2 diabetes patient, previously diagnosed by fasting plasma glucose or by a 2-hour oral glucose tolerance test (OGTT) according to WHO criteria.
2. Age females: 55-70 years (post-menopausal state defined as natural amenorrhea for at least 12 months); age males: 40-70 years.
3. Body Mass Index (BMI): 27-40 kg/m^2.
4. Haemoglobin A1c (HbA1c) < 60 mmol/mol.
5. Type 2 diabetes duration > 3 months and < 10 years.
6. Understand and speak Swedish.

Exclusion criteria were detailed as follows:
1. Anti-hyperglycaemic treatment with glitazones, glucagon-like peptide 1 (GLP-1) analogues or Dipeptidyl peptidase 4 (DPP-4) inhibitors.
2. Antihypertensive therapy with beta-blockers, angiotensin-converting enzyme (ACE) inhibitors and/or angiotensin II receptor blockers (ARBs).
3. Significant microvascular complications such as nephropathy of Glomerular Filtration Rate (GFR) < 60 ml/min/1.73 m2), proliferative retinopathy and symptomatic neuropathy, e.g., postural hypotension.
4. Previous significant vascular disease including angina pectoris and myocardial infarction, cerebral artery disease, e.g., history of transitoric ischaemic attacks and peripheral artery disease with no palpable pulses.
5. Smoking > 10 cigarettes/day and/or use of smokeless tobacco > 1 can per 2 days.
6. Concurrent use of nitrates or nitric oxide (NO) donors, or an apparent risk that there may be a need of such medication.
7. Cardiac failure (New York Heart Association (NYHA) II-IV).
8. Uncontrolled hypertension > 170/105 mm Hg.
9. Apparent electrocardiogram (ECG) pathology indicating current or previous myocardial ischaemia.
10. Males with erectile dysfunction.
11. Haemophilia or a history of bruises or hepatic failure (> 2-fold increase upper limit normal values of aspartate aminotransferase (AST) and alanine aminotransferase (ALT).
12. Hypotension.
13. Treatment with doxazosin.
14. Anything in the contact with the participant that makes the doctor to believe that he/she will be uncompliant to the protocol.

Supplementary Table 1. Medical history reported at screening for the full analysis set population, n=18

Condition	n (%)
**Cardiac disorders**	**3 (16.7%)**
Angina pectoris	2 (11.1%)
Cardiac hypertrophy	1 (5.6%)
**Endocrine disorders**	**3 (16.7%)**
Hyperparathyroidism	1 (5.6%)
Hypothyreosis	1 (5.6%)
Thyroiditis	1 (5.6%)
**Gastrointestinal disorders**	**4 (16.7%)**
GERD	2 (11.1%)
Gastritis	1 (5.6%)
Irritable bowel syndrome	1 (5.6%)
**Immune system disorders**	**2 (11.1%)**
Grass allergy	1 (5.6%)
Rubber sensitivity	1 (5.6%)
**Infections and infestations**	**2 (11.1%)**
Bronchitis	1 (5.6%)
Fungal foot infection	1 (5.6%)
**Injury, poisoning and procedural complications**	**2 (11.1%)**
Wasp sting	1 (5.6%)
Whiplash injury to neck	1 (5.6%)
**Investigations**	**1 (5.6%)**
Opiates	1 (5.6%)
**Metabolism and nutrition disorders**	**7 (38.9%)**
Gout	1 (5.6%)
Hypercholesterolaemia	6 (33.3%)
**Musculoskeletal and connective tissue disorders**	**10 (55.6%)**
Back pain	1 (5.6%)
Coxarthrosis	3 (16.7%)
Foot osteoarthritis	1 (5.6%)
Gonarthrosis	2 (11.1%)
Lumbago	1 (5.6%)
Lumbar disc herniation	1 (5.6%)
Osteoarthrosis	1 (5.6%)
Osteoporosis	1 (5.6%)
Spinal stenosis	1 (5.6%)
**Neoplasms benign, malignant and unspecified**	**1 (5.6%)**
Testis cancer	1 (5.6%)
**Psychiatric disorders**	**1 (5.6%)**
Anxiety	1 (5.6%)
Insomnia	1 (5.6%)
**Renal and urinary disorders**	**1 (5.6%)**
Overactive bladder	1 (5.6%)
**Respiratory, thoracic and mediastinal disorders**	**3 (16.7%)**
Asthma	1 (5.6%)

Sleep apnea	2 (11.1%)
**Surgical and medical procedures**	**2 (11.1%)**
Circumcision	1 (5.6%)
Prostate surgery	1 (5.6%)
**Vascular disorders**	**10 (55.6%)**
Aortic stenosis	1 (5.6%)
DVT of legs	1 (5.6%)
Hypertension	10 (55.6%)

GERD, gastroesophageal reflux disease; DVT, deep venous thrombosis

Supplementary Table 2. Concomitant medications of the full analysis set population, n=18

	**Participants with medications**<br><br>**n (%)**
**Any medication**	**17 (94.4)**
**Anti-hyperglycaemic agents**	**17 (94.4)**
Metformin	16 (88.9)
Glimepiride	2 (11.1)
Glipizide	1 (5.6)
Empagliflozin	2 (11.1)
**Anti-hypertensive treatment**	**8 (44.4)**
**Treatment for hypercholesterolaemia with statins**	**8 (44.4)**
**Respiratory organs**	**4 (22.2)**
Cetirizine	1 (5.6)
Loratadine	1 (5.6)
Cough and cold preparations	1 (5.6)
Mometasone	1 (5.6)
Xylometazoline	2 (11.1)
**Anti-infectives for systemic use**	**1 (5.6)**
Flucloxacillin	1 (5.6)
**Blood and blood forming organs**	**1 (5.6)**
Apixaban	1 (5.6)
**Cardiovascular system**	**8 (44.4)**
Amlodipine	3 (16.7)
Bendroflumetiazide	2 (11.1)
Furosemide	1 (5.6)
Diuretics and potassium-sparing agents in combination	3 (16.7)
**Dermatologicals**	**1 (5.6)**
Econazole	1 (5.6)
**Alimentary tract and metabolism**	**10 (55.6)**
Potassium chloride	2 (11.1)
Loperamide	1 (5.6)
Magnesium (different salts in combination)	1 (5.6)
Macrogol in combination with electrolytes	1 (5.6)
Sodium lauryl sulfoacetate, incl. combination	1 (5.6)
Sodium picosulfate	1 (5.6)
Omeprazol	5 (27.8)
Pantoprazol	1 (5.6)
**Nervous system**	**11 (61.1)**
Gabapentin	1 (5.6)
Codeine and paracetamol	2 (11.1)
Sodium salicylate	1 (5.6)
Oxycodone	3 (16.7)
Paracetamol	10 (55.6)

Paroxetine	1 (5.6)
**Musculoskeletal system**	**10 (55.6)**
Diclofenac	5 (27.8)
Etoricoxib	1 (5.6)
Ibuprofen	2 (11.1)
Naproxen	2 (11.1)
**Sensory organs**	**1 (5.6)**
Sodium cromoglycate	1 (5.6)

Supplementary Table 3. Type of adverse events registered in the safety population

	Participants with events on placebo, n=22 n (%)	Participants with events on tadalafil, n=21 n (%)	*p*-value
**Any event**	**14 (63.6%)**	**19 (90.5%)**	0.069
**Cardiac disorders**	**1 (4.5%)**	**0 (0%)**	>0.99
Palpitations	1 (4.5%)	0 (0%)	
**Eye disorders**	**0 (0%)**	**3 (14.3%)**	0.11
Eye pain	0 (0%)	1 (4.8%)	
Eye swelling	0 (0%)	1 (4.8%)	
Vision blurred	0 (0%)	1 (4.8%)	
**Gastrointestinal disorders**	**6 (27.3%)**	**15 (71.4%)**	**0.006**
Abdominal pain	0 (0%)	1 (4.8%)	
Abdominal pain upper	0 (0%)	1 (4.8%)	
Anal fistula	0 (0%)	1 (4.8%)	
Constipation	1 (4.5%)	1 (4.8%)	
Diarrhoea	1 (4.5%)	4 (19.0%)	
Dyspepsia	1 (4.5%)	7 (33.3%)	
Gastritis	0 (0%)	2 (9.5%)	
Gastroesophageal reflux disease	2 (9.1%)	5 (23.8%)	
Haematochezia	0 (0%)	1 (4.8%)	
Haemorrhoids	0 (0%)	1 (4.8%)	
Nausea	2 (9.1%)	0 (0%)	
**General disorders and administration site conditions**	**1 (4.5%)**	**6 (28.6%)**	**0.046**
Chest pain	0 (0%)	1 (4.8%)	
Fatigue	1 (4.5%)	1 (4.8%)	
Oedema peripheral	0 (0%)	1 (4.8%)	
Pyrexia	0 (0%)	3 (14.3%)	
**Immune system disorders**	**1 (4.5%)**	**0 (0%)**	>0.99
Seasonal allergy	1 (4.5%)	0 (0%)	
**Infections and infestations**	**7 (31.8%)**	**6 (28.6%)**	>0.99
Gastroenteritis viral	1 (4.5%)	0 (0%)	
Genital infection fungal	1 (4.5%)	0 (0%)	
Influenza	1 (4.5%)	0 (0%)	
Nasopharyngitis	5 (22.7%)	4 (19.0%)	
Pyelonephritis	0 (0%)	1 (4.8%)	
Rhinitis	0 (0%)	1 (4.8%)	
Urinary tract infection	0 (0%)	1 (4.8%)	
**Injury, poisoning and procedural complications**	**3 (13.6%)**	**1 (4.8%)**	0.61
Humerus fracture	0 (0%)	1 (4.8%)	
Procedural pain	2 (9.1%)	0 (0%)	
Radius fracture	1 (4.5%)	0 (0%)	
**Metabolism and nutrition disorders**	**1 (4.5%)**	**0 (0%)**	>0.99
Decreased appetite	1 (4.5%)	0 (0%)	
**Musculoskeletal and connective tissue disorders**	**8 (36.4%)**	**15 (71.4%)**	**0.033**
Arthralgia	1 (4.5%)	5 (23.8%)	
Arthritis	1 (4.5%)	0 (0%)	
Back pain	1 (4.5%)	5 (23.8%)	
Muscle spasms	1 (4.5%)	2 (9.5%)	
Musculoskeletal stiffness	1 (4.5%)	1 (4.8%)	

Myalgia	1 (4.5%)	4 (19.0%)	
Neck pain	1 (4.5%)	1 (4.8%)	
Pain in extremity	1 (4.5%)	4 (19.0%)	
**Nervous system disorders**	**5 (22.7%)**	**12 (57.1%)**	**0.031**
Burning feet syndrome	0 (0%)	1 (4.8%)	
Dizziness	0 (0%)	1 (4.8%)	
Head discomfort	0 (0%)	1 (4.8%)	
Headache	2 (9.1%)	10 (47.6%)	
Migraine	1 (4.5%)	1 (4.8%)	
Paraesthesia	3 (13.6%)	1 (4.8%)	
**Psychiatric disorders**	**0 (0%)**	**2 (9.5%)**	0.23
Insomnia	0 (0%)	2 (9.5%)	
**Respiratory, thoracic and mediastinal disorders**	**1 (4.5%)**	**5 (23.8%)**	0.095
Dyspnoea	0 (0%)	2 (9.5%)	
Epistaxis	0 (0%)	1 (4.8%)	
Nasal congestion	0 (0%)	2 (9.5%)	
Oropharyngeal pain	0 (0%)	2 (9.5%)	
Productive cough	1 (4.5%)	0 (0%)	
**Skin and subcutaneous tissue disorders**	**1 (4.5%)**	**2 (9.5%)**	0.61
Hyperhidrosis	0 (0%)	1 (4.8%)	
Skin irritation	1 (4.5%)	0 (0%)	
Swelling face	0 (0%)	1 (4.8%)	
**Surgical and medical procedures**	**2 (9.1%)**	**1 (4.8%)**	>0.99
Dental implantation	1 (4.5%)	0 (0%)	
Hip arthroplasty (SAE)	1 (4.5%)	0 (0%)	
Medical device removal	0 (0%)	1 (4.8%)	
**Vascular disorders**	0 (0%)	**2 (9.5%)**	0.23
Flushing	0 (0%)	2 (9.5%)	

CLINICAL STUDY PROTOCOL

Effects on insulin resistance with the phosphodiesterase-5 inhibitor tadalafil in type 2 diabetes – a double-blind, placebo-controlled crossover study

**Study Code:**	MAKROTAD
**EudraCT Number:**	2015-000573
**Version Number:**	2.1
**Date:**	2015-09-14

Sponsor:

Professor Per-Anders Jansson
Wallenberg Laboratory, Bruna Stråket 16
Dep of Molecular and Clinical Medicine, University of Gothenburg, Sahlgrenska University Hospital, S-413 45 Gothenburg, SWEDENDep of Molecular and Clinical Medicine, University of Gothenburg, Sahlgrenska University Hospital, S-413 45 Gothenburg, SWEDEN
E-mail: per-anders.jansson@medic.gu.seE-mail: per-anders.jansson@medic.gu.se
Cell: +46 (0)70 203-30 10Cell: +46 (0)70 203-30 10

Investigator:

Professor Per-Anders Jansson
Wallenberg Laboratory, Bruna Stråket 16
Dep of Molecular and Clinical Medicine, University of Gothenburg, Sahlgrenska University Hospital, S-413 45 Gothenburg, SWEDENDep of Molecular and Clinical Medicine, University of Gothenburg, Sahlgrenska University Hospital, S-413 45 Gothenburg, SWEDEN
E-mail: per-anders.jansson@medic.gu.seE-mail: per-anders.jansson@medic.gu.se
Cell: +46 (0)70 203-30 10Cell: +46 (0)70 203-30 10

Synopsis

Title: Effects on insulin resistance by using the phosphodiesterase-5 (PDE5) inhibitor tadalafil in type 2 diabetes patients – a double-blind, placebo-controlled crossover study

Study period: The study will be conducted on one study site. Total study length 20 weeks, enrollment period 3-4 years, treatment period 2015-2018.

Hypothesis: The phosphodiesterase-5 inhibitor tadalafil is a useful drug to treat insulin resistance in type 2 diabetes patients.

Aim: In this project, we aim to continue our program on PDE5 inhibition by evaluating effects on insulin resistance, including glucose metabolism and subclinical inflammation, after a 6-week administration of tadalafil in T2D patients.

Primary objective: Our primary objective is to study the effect of tadalafil compared with placebo on insulin sensitivity during a euglycemic hyperinsulinemic clamp.

Secondary objectives regarding the effects of tadalafil:

1) to study glucose homeostasis by measuring circulating glucose and insulin concentrations, arginine-induced insulin secretion and transendothelial transport of insulin in adipose tissue and skeletal muscle as measured by the microdialysis technique
2) to study effects on circulating inflammatory markers and pathways including expression of c-Jun N-terminal Kinase (JNK) in microvascular endothelial cells (MVEC) obtained from subcutaneous needle biopsies;
3) to study endothelial function by performing blood flow measurements and peripheral arterial tonometry (EndoPAT).

Study outline: A double-blind, placebo-controlled crossover trial. One clinical site.

Treatment and dosage: Twenty-five T2D patients will be recruited and randomized to per oral intake of tadalafil 20 mg o.d. for six weeks and after a wash-out period of eight weeks intake of placebo for another six weeks, or vice versa.

Study population: The study will include 25 type 2 diabetes patients (men and post-menopausal women) recruited by advertisement or via Primary Health Care Centers in the Region Västra Götaland. For safety reason administration of nitroglycerin must be avoided by the participants due to risk of severe hypotension.

Primary outcome variables and examinations: At the end of each 6 week treatment period a 3-hour glucose clamp (insulin sensitivity), subcutaneous needle biopsies (cell signaling studies) as well as muscle and subcutaneous microdialysis (transendothelial insulin transport) will be performed. Endothelial function tests and arginin stimulation of insulin secretion tests will be performed after 3 weeks in each treatment arm. Blood (metabolic and inflammatory markers), urine (albuminuria), and faeces samples (microbiota composition) will also be collected during the study.

TABLE OF CONTENTS

1. Introduction 7
1.1. Background 7
1.2. Rationale for conducting this study 9
1.3. Research hypothesis 10
1.4. Risk/Benefit evaluation 10
1.5. Ethical assessment 11
2. Study Objectives and Endpoints 11
2.1. Primary objective 11
2.2. Secondary objectives 11
3. Study Design and Procedures 11
3.1. Overall study design and flow chart 11
4. Study Population 14
4.1. Inclusion criteria 15
4.2. Exclusion criteria 15
4.3. Restrictions 15
4.4. Subject enrolment and randomization 16
4.5. Discontinuation and withdrawal of subjects 16
4.5.1. Premature termination of the study 16
5. Study Treatments 16
5.1. Identity of investigational medicinal products 16
5.1.1. Doses and treatment regimens 16
5.1.2. Labelling 16
5.1.3. Storage and handling 16
5.1.4. Drug accountability and treatment compliance 17
5.2. Blinding 17
5.3. Randomization 17
5.4. Concomitant medication 17
6. Study Measurements and Variables 17
6.1. Primary variable 17
6.2. Secondary variable(s) 17
6.3. Safety variable(s) 18
6.4. Biological sampling procedures 18
6.4.1. Handling, storage and destruction of biological samples 18
7. Safety 18
7.1. Definitions 18
7.1.1. Adverse event (AE) 18
7.1.2. Adverse drug reaction 19
7.1.3. Serious adverse event (SAE) 19
7.1.4. Unexpected and expected adverse events 19
7.1.5. Suspected unexpected serious adverse reaction (SUSAR) 20
7.2. Reporting 20
7.2.1. Adverse event 20
7.2.2. Adverse drug reaction 20
7.2.3. Serious adverse event (SAE) 20

7.2.4. Suspected unexpected serious adverse reaction (SUSAR) ... 20
7.2.5. Adverse Event Collection Period ... 21
7.2.6. Annual safety report ... 21
8. Statistics ... 21
8.1. Sample size calculation ... 21
8.2. Populations ... 21
8.2.1 Full analysis data set (based on the ITT principle) ... 21
8.2.2 Per Protocol (PP) population ... 21
8.2.3 The Safety population ... 22
8.3. General statistical methodology ... 22
8.4. Efficacy Analyses ... 22
8.4.1. Primary efficacy analysis ... 22
8.4.2. Secondary efficacy analyses ... 22
8.5. Demographics and Baseline Characteristics ... 23
8.6. Safety analyses ... 23
8.7. Statistical Analysis Plan ... 23
9. Data Management ... 23
9.1. Recording of data ... 23
10. Quality Control and Quality Assurance ... 24
10.1. Monitoring ... 24
10.2. Audits and inspections ... 24
10.3. Other ... 24
11. Ethics ... 24
11.1. Informed consent ... 24
11.2. Ethics committee ... 25
11.3. Subject data protection ... 25
11.4. Insurances ... 25
12. Protocol Amendments ... 25
13. Report and publications ... 25
14. Study Timetable ... 26
14.1. Definition of “End of study” ... 26
15. List of References ... 26
16. Signatures ... 27

LIST OF ABBREVIATIONS

Abbreviation	Explanation
AE	adverse event
APL	apoteket produktion & laboratorier AB
ATBF	adipose tissue blood flow
cGMP	cyclic guanosine monophosphate
CRP	c-reactive protein
ET-1	endothelin-1
EndoPAT	peripheral arterial tonometry
FBF	forearm blood flow
GDR	glucose disposal rate
HUVEC	human umbilical vein endothelial cells
ICAM-1	intercellular adhesion molecule 1
IRS	insulin resistance syndrome
JNK	c-Jun N-terminal kinase
MD	microdialysis
MVEC	microvascular endothelial cells
NO	nitric oxide
PDE5	phosphodiesterase-5
SAE	serious adverse event
SUSAR	suspected unexpected serious adverse reaction
Tadalafil	PDE5 inhibitor marketed in pill form for treating erectile dysfunction under the name Cialis®
TNFα	tumor necrosis factor alpha
T2D	type 2 diabetes mellitus
VCAM-1	vascular cell adhesion molecule 1

1. INTRODUCTION

1.1. Background

Disease
Obesity is an important public health problem associated with inflammation, cardiovascular disease, metabolic syndrome, and type 2 diabetes (T2D). The incidence of T2D and its complications has been growing rapidly (worldwide about 400 million T2DM patients by 2030) and is reaching epidemic proportions.

Treatment
Current blood glucose lowering strategies are based on amelioration of insulin sensitivity and insulin secretion [1], but long-term this approach seems insufficient to keep T2D patients in good metabolic control [2]. Clearly pharmacological treatment of the disease needs to be improved.

Microvascular dysfunction and insulin resistance
Several pioneering studies indicate strong associations between microvascular dysfunction and insulin resistance [3, 4]. It is well established that impaired action of insulin in the vasculature of insulin-resistant patients reduces synthesis of nitric oxide (NO) in endothelial cells contributing to microvascular dysfunction [5]. Hence, NO-donating agents and compounds that modulate bioactivity of endogenously synthesized NO may represent powerful tools to improve insulin sensitivity in insulin-resistant states [6].

Nitric oxide, phosphodiesterase-5 (PDE5) inhibition, endothelial function, and glucose uptake
A previous report showed that methacholine, which induces NO production, has a positive metabolic effect on vascular function and muscle metabolism in rats in vivo [7].We followed up on this observation and reported beneficial effects of methacholine on capillary recruitment and forearm glucose uptake in insulin-resistant obese subjects [8]. Since metacholine is an investigational medicinal product and not suitable for therapeutic use we tested whether PDE5 inhibition may be an alternative approach to increase NO synthesis and ameliorate endothelial dysfunction. We suggest that phosphodiesterase-5 (PDE5) inhibition results in an amplified NO effect (fig 1) and may improve vascular and metabolic function in patients with insulin resistance and T2D. We have shown that a single dose of tadalafil (20 mg) benefits women with T2D. Patients included in this study exhibited immediate positive metabolic effects as shown by increased glucose uptake and capillary recruitment [9]. We have also shown that tadalafil is an acutely acting modulator of glucose uptake in adipose tissue [10].

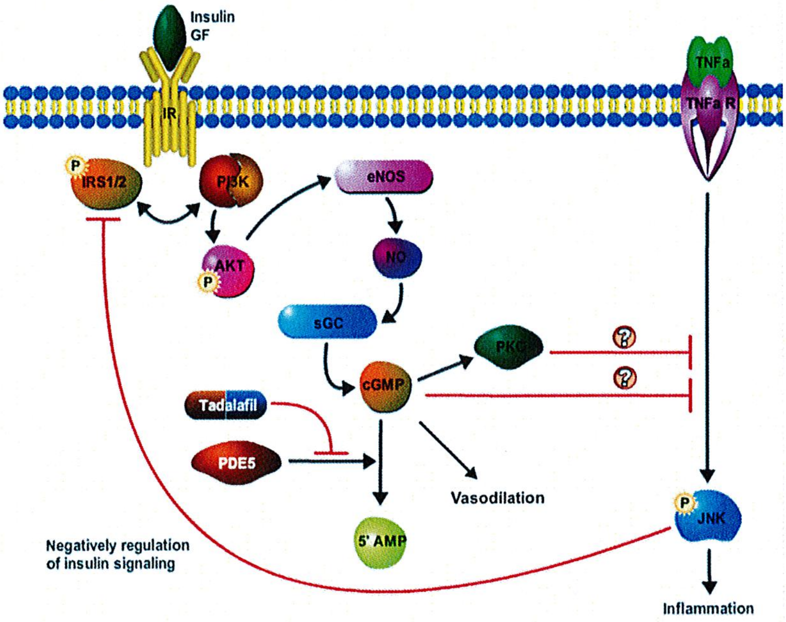

Fig 1. Pathways we will address in this project by testing the therapeutic potential of tadalafil to ameliorate vascular and metabolic insulin resistance in T2D patients.

Effects of tadalafil in insulin-resistant participants

We have provided clear evidences of acute microvascular and metabolic effects of tadalafil in insulin-resistant participants [9-11] but very little is known about the metabolic effects of chronic PDE5 inhibition in insulin-resistant participants [12, 13]. In a US study, patients with the metabolic syndrome were treated for 3 weeks with 10 mg tadalafil o.d. [13]. In this study, PDE5 inhibition improved beta-cell function but showed no effect on insulin sensitivity. To our knowledge there are no available data on the metabolic effects of daily administration of 20 mg tadalafil for 6 weeks in T2D patients, i.e. "chronic" administration. We propose to investigate this important knowledge gap to determine the therapeutic potential of a higher dose of tadalafil to improve different aspects of vascular and metabolic insulin resistance in T2D patients.

Subclinical inflammation in T2D patients

A further aim of this project is to investigate whether tadalafil attenuates subclinical inflammation in T2D patients. It is well established that inflammatory mediators such as tumor necrosis factor alpha (TNFα) play a significant role in promoting insulin resistance [14]. It has also been reported that circulating TNFα levels are associated with skin capillary recruitment in humans [15] indicating a relationship between TNFα and insulin resistance [14, 16]. In addition, TNFα inhibits vasodilator but not vasoconstrictor effects of insulin via activation of c-Jun N-terminal (JNK) in rat skeletal muscle resistance arteries [17]. Further, T2D patients treated for 3 weeks with the PDE5 inhibitor sildenafil showed an improved endothelial function and reduced circulating markers of vascular inflammation, such as endothelin-1 (ET-1), C-reactive protein (CRP), intercellular adhesion molecule 1 (ICAM-1) and vascular cell adhesion molecule 1 (VCAM-1) [12]. In another study, chronic treatment with tadalafil resulted in decreased ET-1 levels and improved endothelial function in men with increased cardiovascular risk [18]. Thus, chronic PDE5 inhibition in T2D patients may decrease

circulating markers of endothelial dysfunction. Therefore, we propose to monitor different circulating markers, blood flow and to test endothelial function by using EndoPAT [19].

Can tadalafil induce anti-inflammatory effects in human microvascular endothelial cells?
We shall also perform experiments in subcutaneous microvascular endothelial cells (MVEC) to investigate whether tadalafil attenuates the inflammatory JNK signaling pathway, promotes insulin signaling via phosphorylation of AKT (fig 1), insulin uptake and its transport across the capillary wall (fig 1) [20]. Data from studies in mice suggest that diet-induced obesity concomitantly reduces vascular NO-cGMP signaling and promotes adipose tissue inflammation and that intervention with PDE5 inhibition reverses the inflammatory state [21]. There are also observations showing that sildenafil restricts vascular inflammation and insulin resistance in mice fed a high fat diet [22]. Based on these results reported from the Seattle group [20, 21] and our preliminary data we would like to investigate whether tadalafil may induce similar anti-inflammatory effects in human microvascular endothelial cells (MVEC) in T2D patients.

A putative novel mechanism that convey anti-inflammatory effects via JNK.
We propose a putative novel mechanism by which the amplified NO-cGMP dependent protein kinase (PKG) signaling pathway may convey anti-inflammatory effects via JNK. The known interplay between low activity in the NO/cGMP/PKG pathway and diet induced vascular inflammation and the observations that TNFα signals via JNK to increase ET-1 levels, makes the purposed chronic study with tadalafil highly relevant [17, 22, 23]. Interestingly, our preliminary data in vitro shows that tadalafil suppresses TNFα-induced inflammation in HUVEC shown as down-regulation of ET-1 expression and tadalafil may also decrease activation of JNK.

1.2. Rationale for conducting this study

Current blood glucose lowering strategies are based on amelioration of insulin sensitivity and insulin secretion[1], but long-term this approach seems insufficient to keep T2D patients in good metabolic control [2]. New pharmacological treatment strategies are therefore needed.

Several pioneering studies indicate strong associations between microvascular dysfunction and insulin resistance [3, 4]. Impaired action of insulin in the vasculature of insulin-resistant patients reduces synthesis of nitric oxide (NO) in endothelial cells contributing to microvascular dysfunction [5]. Hence, NO-donating agents and compounds that modulate bioactivity of endogenously synthesized NO may represent powerful tools to improve insulin sensitivity in insulin-resistant states [6].

In this project, we will investigate the metabolic consequences of an amplified NO-cGMP signaling induced by chronic PDE5 inhibition in insulin-resistant T2D patients, in particular the effects on glucose metabolism. This is the first time PDE5 inhibition induced by the highest recommended dose of tadalafil is given once daily for 6 weeks to T2D patients and followed by assessment of insulin sensitivity by a euglycemic hyperinsulinemic clamp.

This is a novel approach and if we demonstrate improved insulin action and attenuated subclinical inflammation in T2D patients showing different facets of the insulin-resistance syndrome (IRS) that would also be the advent of a new principle to prevent cardiovascular disease in these patients. The clinical impact of a finding like this would be substantial for the future treatment of both glucose

homeostasis impairment and endothelial dysfunction in type 2 diabetes. Finally, we will shed more light on the putative role of the NO-cGMP-JNK pathway for amelioration of subclinical inflammation in the microcirculation.

1.3. Research hypothesis

We have shown that selective inhibition of PDE5 by tadalafil has immediate beneficial effects on peripheral microcirculation, forearm glucose uptake and capillary recruitment and in addition to that suppression of inflammatory markers in T2D patients. We therefore hypothesize that tadalafil may be a useful drug to treat different aspects of insulin resistance.

1.4. Risk/Benefit evaluation

Benefits:
There are no major benefits for subjects participating in the study. However, participants may have improved metabolic control during the study. Moreover, a medical examination is conducted and the participants have the opportunity to make contact with the study team around the clock.

Participants are involved in contributing to that new information is generated about a potential new treatment for T2D.

Risks:
A major disadvantage of participating in the study is that the subjects commit themselves for a 20 week study, including 7 research visits. The study includes 2 arduous days with examinations with several punctures of skin and veins (visit 4 and 7). In addition, subjects commit to taking a study tablet daily for 12 weeks.

Tadalafil is approved for treatment of erectile dysfunction and like all medicines, this medicine can cause side effects, although not everybody gets them. These effects are usually mild or moderate in nature.

Common (affects 1 to 10 of 100 patients) side effects are: headache, back pain, muscle pain, pain in the limbs, facial flushing, nasal congestion, indigestion and acid reflux.
Uncommon (affects 1 to 10 out of 1000 patients) side effects are: dizziness, stomach pain, blurred vision, eye pain, increased sweating, difficulty breathing, bleeding from the penis, presence of blood in semen and/or urine, palpitations, rapid heartbeat, high blood pressure, low blood pressure, nosebleeds and tinnitus.
Rare (affects 1 to 10 of 10000 patients) side effects are: fainting, seizures and passing memory loss, swelling of the eyelids, red eyes, sudden decrease or loss of hearing, and rash.

An ID-card is given to the subject with information how to get in contact with the study team in case of emergency. There is always one doctor in the team available to answer questions if a subject experiences new symptoms that could be a side-effect of the study drug.

For safety reasons administration of nitroglycerin must be avoided in this study, due to risk of severe hypotension. Subjects with such medication are therefore excluded, see exclusion criteria.

The treatment of T2D patients with one 20 mg dose of tadalafil has been done previously [9, 10] However, in this study the treatment is longer i.e. 6 weeks. No other side effects than already reported in previous studies are expected.

Participation in the study will not affect the long-term treatment of the study participants. However, short-term impact on the treatment may occur when certain medications must be omitted before examinations. Participation in the study will therefore be communicated to the treating physician.

1.5. Ethical assessment

Although the study medicine have known risk factors and 2 whole days of examinations are included in the study protocol, the risks of the treatment and the examinations are considered less serious and judged less than the future benefits of a new treatment for patients with T2D.

2. STUDY OBJECTIVES AND ENDPOINTS

2.1. Primary objective

To evaluate the effect of daily administration of 20 mg tadalafil for 6 weeks ("chronic" treatment) on insulin sensitivity in muscle by assessing glucose disposal rate during a 3-hour euglycemic hyperinsulinemic glucose clamp (120 $mU/m^2/min$) in T2D patients.

2.2. Secondary objectives

To evaluate the effect of chronic tadalafil treatment on glucose metabolism and beta-cell function as measured by mean blood glucose (HbA1c), circulating glucose and insulin concentrations, and arginine-induced insulin secretion.

To evaluate whether chronic tadalafil administration down-regulates circulating inflammatory markers and increases interstitial insulin and lactate concentrations in insulin-sensitive tissues and if this could be coupled to signaling in the insulin/IGF-1 and cGMP-PKG-JNK pathways in microvascular endothelial cells (MVEC).

To evaluate whether chronic tadalafil administration improves endothelial function in peripheral arteries.

3. STUDY DESIGN AND PROCEDURES

3.1. Overall study design and flow chart

This is a phase **II**, randomized, double-blind, placebo-controlled crossover study (fig 2). A crossover design is chosen to reduce the influence of confounding covariates when each subject serves as his or her own control. This design is also statistically efficient and requires fewer subjects. There are seven study visits and the subjects will be included for 20 weeks in the study.

Before visits 2, 3, 4, 5, 6 and 7 subjects will be fasting overnight and informed not to take their ordinary medication in the morning. Furthermore, three days prior to a visit (visit 2-7) the

participating subjects will not take their study drug, anti-diabetic or anti-hypertensive medication. In addition, medication with statins and/or ASA will be suspended 1 week before visit 2-7.

The randomization to start with the study drug or placebo will be conducted by Apoteket Produktion & Laboratorier AB, APL. The study drug and the placebo will be taken as tablets and to assure that the study is double-blind, placebo and tadalafil tablets are made visually indistinguishable.

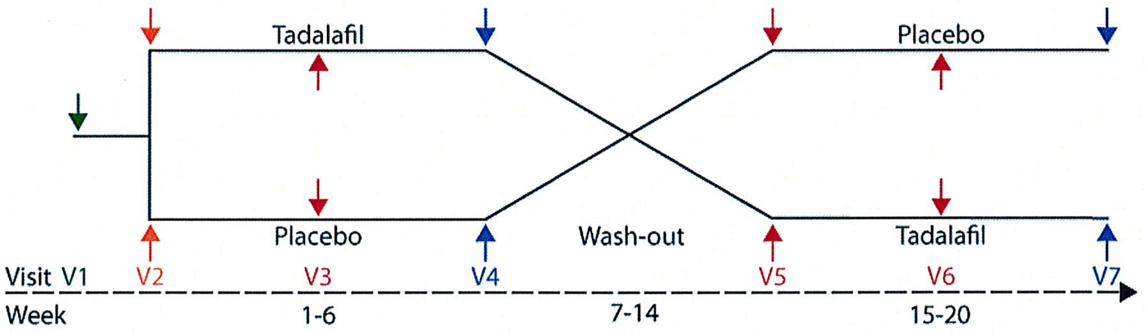

Fig 2. Overview of study protocol. Visit 1: Screening: anthropometry, ECG, blood- and urine sampling. Informed consent. Visit 2: Anthropometry, blood- and urine sampling, EndoPAT, randomization to start-treatment. Each period lasts 6 weeks and tadalafil 20 mg or placebo is taken orally o.d. Visit 3 and 6: Anthropometry, blood- and urine sampling, EndoPAT as well as an arginine-test. Visit 4 and 7: Glucose clamp, subcutaneous needle biopsy procedure, microdialysis and blood flow measurements.

At the screening visit signed informed consent is obtained and blood sampling for routine check of blood-, liver-, electrolyte- and metabolic status at the accredited Central Laboratory at Sahlgrenska University Hospital is performed. A physical exam is performed and a medical history is taken by the study physician. The inclusion/exclusion criteria are reviewed. If the subject fulfils the inclusion- and exclusion criteria randomization will take place at visit 2 (Further details in fig 2). Subjects get a diary to fill out if new symptoms appear and whether the tablet was taken. A new diary is handed out and the old one is collected and reviewed by the study team at each visit. Importantly, an ID-card is given to the subject with information how to get in contact with the study team in case of emergency. There is always one doctor in the team available to answer questions if a subject experiences new symptoms that could be a side effect of the study drug.

An arginine-test will be performed at visit 3 and 6. After baseline sampling endothelial function will be assessed with EndoPAT, a non-invasive procedure measuring dilation and pulsations in the index finger [19]. Next, body composition will be analysed by bioimpedance. After that an intravenous pulse of 5 g arginine is given at time 0 followed by p-glucose, s-insulin, and c-peptide sampling 2, 3, 4, 5, 7, 10, 25 and 30 min following the injection [24].

The glucose clamp day at visit 4 and 7 will start at 08:00 h after an overnight fast, and the participants will be examined in the supine position in a room kept at 26±1°C. A catheter will be inserted into a

deep-vein in the antecubital fossa of the non-dominant arm, for blood sampling. Another catheter will be placed in a forearm superficial vein for 5-min sampling and covered by a heating pad.

Then, an abdominal subcutaneous needle biopsy is performed under local anesthesia, and specimens will be biobanked. The obtained specimens are used for determination of fat cell size, isolation of microvascular endothelial cells for further studies in primary cultures and isolated fat cells are treated to get lysates comparing basal and insulin stimulation on activation of key proteins in the insulin signaling pathway [25, 26]. Then, 300 mg adipose tissue is immediately put in a tube with RNA-Later and stored at -80°C for later mRNA extraction [26]. Next, forearm blood flow (FBF) will be measured by venous occlusion plethysmography (Hokanson, Bellevue, WA) and adipose tissue blood flow (ATBF) will be estimated by Xenon-clearance [11]. Blood pressure will be measured using a semi-automated oscillometric device (Omron CP-705; Omron Healthcare, Milton Keynes, UK). At baseline, three different blood pressure measurements will be performed and then every 30 min during the glucose clamp.

Two microdialysis (MD) catheters (20 kD molecular mass cutoff, CMA Microdialysis AB; CMA, Stockholm) will be inserted into the brachioradialis muscle of the dominant arm. For subcutaneous microdialysis measurements of insulin and lactate two ultrafiltrating probes and two Cuprophane membranes are placed in the periumbilical subcutaneous tissue as previously described [9, 11]. Dialysates and blood samples are collected every 15-30 min during the day: from 30 min prior to start of the clamp and until 4 hours after the insulin infusion was commenced for monitoring of subcutaneous interstitial lactate concentrations and estimation of the interstitial/circulating insulin ratio, an index of the endothelial barrier [11]. Finally, between 180 and 240 min after start of the clamp we calibrate the insulin catheters in situ via collection of dialystaes for later determination of the interstitial concentration of the reference substance inulin [11]. The principle of microdialysis has been described previously [10] and glucose uptake (μmol/min*100 g) is estimated according to Fick's principle: uptake = muscle blood flow x (A-V glucose).

Glucose and insulin are infused at 120 mU/m2/min due to the severe insulin resistance usually prevailing in T2D patients. Arterialized venous plasma glucose is checked every 5 min aiming for a plasma glucose concentration at 5.5 mmol/l. The last 30 min of the 3-hour insulin infusion are considered as steady state, and the glucose disposal rate (mg/kg/min) during steady state is the primary endpoint in the study.

Faeces samples will be collected during the day before visit 2, 4 and 7 to study the intestinal microbiota in relation to study drug. Tools will be handed out to the research subject and faeces equivalent to one finger tip will be placed with a stick in a transport tube which is then placed in the freezer at home before the research subject brings the tube to the laboratory for final storage at - 80 ° C. The research subjects are asked to collect faeces from both a morning and an evening defecation.

Activity	Screeening visit	Visit 2, week 1	Visit 3	Visit 4, Week 6	Visit 5, week 14	Visit 6	Visit 7, week 20
Study information	x						
Informed consent	x						
Demography	x						
Blood sampling (20ml)	x						
Physical exam	x						
Medical history	x						
Inclusion/exclusion criteria	x						
Blood sampling (fasting, 20-210 ml)		x	x	x	x	x	x
Urine sampling	x	x	x	x	x	x	x
Faeces sample		x		x			x
Anthropometry - weight, length, waist, hip	x	x	x	x	x	x	x
ECG	x						
Randomization		x					
EndoPAT		x	x		x	x	
Arginine-test			x			x	
Fat biopsy				x			x
Glucose clamp				x			x
Venous occlusion plethysmography				x			x
Xenon clearance				x			x
Subcutaneous and muscle microdialysis				x			x
Body composition analysis (bioimpedance)			x			x	
Concomitant medication		x	x	x	x	x	x
Adverse events		x	x	x	x	x	x
Diary exchange and review			x	x	x	x	x
Study termination							x

4. STUDY POPULATION

The study will include 25 subjects with type 2 diabetes of both gender, recruited by advertisement or from Primary Health Care Centres in the Region Västra Götaland.

4.1. Inclusion criteria

To be eligible for the study, the participants have to fulfill the following inclusion criteria:

1) T2D patient, previously diagnosed by fasting or 2-hr OGTT plasma glucose levels
2) Age females: 55-70 yrs (post-menopausal state defined as natural amenorrhea for at least 12 months); Age males: 40-70 yrs
3) BMI: 27-40 kg/m^2
4) HbA1c < 60 mmol/mol
5) Type 2 diabetes duration > 3 months and < 10 yrs
6) Understand and speak Swedish

4.2. Exclusion criteria

The following exclusion criteria have been decided:

1) Diabetes treatment with glitazones, GLP-1 analogues or DPP-IV inhibitors
2) Anti-hypertensive therapy with beta-blockers, ACE-inhibitors and/or angiotensin-II receptor blockers
3) Significant microvascular complications e.g. nefropathy (GFR<60), proliferative retinopathy and symptomatic neuropathy e.g. postural hypotension
4) Previous significant vascular disease including angina pectoris and myocardial infarction, cerebral artery disease e.g. history of transitoric ischemic attacks and peripheral artery disease with no palpable pulses
5) Smoking > 10 cig/day and/or smokeless tobacco > one can per 2 days
6) Concurrent use of nitrates or NO donors, or an apparent risk that there may be a need of such medication
7) Cardiac failure (stages NYHA II-IV)
8) Uncontrolled hypertension > 170/105 mm Hg
9) Apparent ECG-pathology indicating current or previous myocardial ischemia;
10) Males with erectile dysfunction
11) Hemophilia or a history of bruises or hepatic failure (> 2-fold increase upper limit normal values of ASAT/ALAT)
12) Hypotension
13) Treatment with doxazosin
14) Anything in the contact with the patient that makes the doctor to believe that he/she will be uncompliant to the protocol.

4.3. Restrictions

For safety reasons administration of nitroglycerin must be avoided due to risk of severe hypotension.

Patients who wish to participate in the study should consult with their treating physician about the suitability of doing so, as participants are informed not to take their ordinary medication up to 3 days before study visits. For more information se the second paragraph of 3.1

4.4. Subject enrolment and randomization

Subject eligibility will be established before enrolment/treatment randomization. Subjects will be enrolled/randomized strictly sequentially, as subjects are eligible for randomization. If a subject discontinues from the study, the subject number will not be reused, and the subject will not be allowed to re-enter the study.

4.5. Discontinuation and withdrawal of subjects

Subjects are free to discontinue their participation in the study at any time without prejudice to further treatment. The subjects may be withdrawn from the study at the discretion of the investigator due to safety concerns or if judged non-compliant with study procedures. In either case, serious adverse events will be followed up. Other reasons for discontinuing a subject are incorrect enrolment and subjects lost to follow-up.

4.5.1. Premature termination of the study

The sponsor may decide to stop the trial or part of the trial at any time. If a trial is prematurely terminated or suspended, the investigator should promptly inform the subjects and ensure appropriate therapy and follow-up. Furthermore, the investigator should promptly inform the Ethics Committee and provide a detailed written explanation. The regulatory authority should be informed according to national regulations.

5. STUDY TREATMENTS

5.1. Identity of investigational medicinal products

The study medication is approved for erectile dysfunction.

- Cialis (active substance tadalafil), Eli Lilly Sweden AB
  Box 721, 169 27 Solna, Sweden.
  Phone: 08-737 88 00, E-mail: info_sweden@lilly.com
- Placebo, Eli Lilly Sweden AB

The investigational medicinal product and the placebo are provided by the manufacturer (Eli Lilly).

5.1.1. Doses and treatment regimens

Cialis (active substance tadalafil), daily administration of 20 mg tadalafil in tablets for 6 weeks. Placebo, daily administration of tablets for 6 weeks.

5.1.2. Labelling

Labelling as will be conducted by Apotek Produktion & Laboratorier AB (APL) according to regulations.

5.1.3. Storage and handling

The tablets will be stored according to product-specific information at the study site in a locked cabinet.

5.1.4. Drug accountability and treatment compliance

The investigator or his/her designated representatives will give study drug only to subjects enrolled in the study. To be able to follow the treatment compliance the subjects is asked to note whether the tablet was taken in a study diary. The diary is reviewed and exchanged at every visit.

5.2. Blinding

Both Cialis (tadalafil) and the placebo are manufactured by Eli Lilly, and the study drug and the placebo are visually indistinguishable.

5.3. Randomization

Randomization is conducted by APL.

5.4. Concomitant medication

Medications that are considered necessary for the subject's safety and well-being may be given at the discretion of the investigators unless specified in the exclusion criteria. Concomitant medication will be recorded in the Case Report Form. Treatment with potent CYP3A4 inhibitors (ie aminodarone, cyclosporine, clarithromycin, dexamethasone, erythromycin, ethinyl estradiol, fluconazol, ketoconazole, metronidazol, norfloxacin, omeprazol telithromycin, or chloramphenicol) should be avoided.

6. STUDY MEASUREMENTS AND VARIABLES

6.1. Primary variable

Glucose disposal rate (GDR equals M-value, mg/kg body weight/min) during the glucose clamp is the main outcome parameter.

6.2. Secondary variable(s)

- Glucose metabolism and beta-cell function as measured by mean blood glucose (HbA1c), circulating glucose and insulin concentrations, and arginine-induced insulin secretion
- Levels of circulating inflammatory markers in blood will be evaluated by using accredited methods at the Central Laboratory at Sahlgrenska University Hospital, Göteborg or by purchasing commercially available ELISA kits
- Levels of interstitial insulin will be evaluated by using commercially available kits from Mercodia AB, Uppsala, Sweden
- Interstitial levels of lactate will be evaluated in dialysates by using the CMA 600 analyzer
- Signaling in the insulin/IGF-1 and cGMP-PKG-JNK pathways in MVEC will be evaluated by immunoprecipitations, immunoblotting and qPCR at the Wallenberg laboratory
- Endothelial function in peripheral arteries will be evaluated by EndoPAT

- Intestinal microbiota in faeces samples will be analyzed at the Wallenberg laboratory

6.3. Safety variable(s)

Unsolicited adverse events (AEs) and serious adverse events (SAEs) will be assessed over the entire duration of the study. Rates, grade and relationship with treatment will be analyzed for each of the treatments groups.

6.4. Biological sampling procedures

6.4.1. Handling, storage and destruction of biological samples

Dialysates and blood samples will be stored in an IVO registered biobank (biobank nr 890 at Sahlgrenska biobank, Sahlgrenska University Hospital) until further analysis. In total approximately 580 ml blood and 2-3 g fat tissue will be collected.

Glucose concentrations in plasma (from arterialized and deep-venous blood) and microdialysates will be determined enzymatically on a CMA 600 Microdialysis Analyser (CMA, Stockholm, Sweden).

Insulin, c-peptide and circulating inflammatory markers (eg Endothelin-1, hs-CRP) will be evaluated by using accredited methods at the Central Laboratory at Sahlgrenska University Hospital, Göteborg or by purchasing commercially available ELISA kits.

The extraction procedure to get MVEC and the medium used are described in our method paper from 2009 (26). We have gained more and more experience how the human MVECs behave in primary culture allowing us to explore inflammatory and insulin/IGF-1 signaling pathways in these cells.

Immunoprecipitations, immunoblotting and qPCR are routine methods at the Wallenberg laboratory.

Feces samples will be collected by the subject at their home in a provided stool collection tube. Subjects will be instructed to keep the sample frozen until delivered to the clinic. Samples will be stored at -80 until analysis.

7. SAFETY

The investigator will monitor each subject for evidence of adverse events (AEs) on a routine basis throughout the study. The investigator will assess and record any AE in detail including the date of onset, description, severity, duration and outcome, relationship of the AE to study drug, and any action(s) taken. AEs, whether in response to a query, observed by site personnel, or reported spontaneously by the subject will be recorded. All AEs will be followed until a satisfactory conclusion.

7.1. Definitions

7.1.1. Adverse event (AE)

Any untoward medical occurrence in a patient or clinical investigation subject administered a pharmaceutical product and which does not necessarily have a causal relationship with this treatment. An adverse event (AE) can therefore be any unfavourable and unintended sign (including an abnormal

laboratory finding), symptom, or disease temporally associated with the use of a medicinal (investigational) product, whether or not related to the medicinal (investigational) product.

Causality

The investigator is responsible for determining whether there is a causal relationship between an AE and the use of a medicinal product.

Severity

In addition to assessing the relationship of the administration of the investigational product(s) to adverse events, an assessment is required of the intensity (severity) of the event. The following over-all classifications should be used:

Mild (Grade 1): An adverse event that is relatively mild and transient in nature, but can be an annoyance, and does not interfere with normal activities.
Moderate (Grade 2): An adverse event that may be uncomfortable but is not hazardous to health. It may be sufficiently discomforting to interfere with normal activities but does not completely prevent them.
Severe (Grade 3): An adverse event that is incapacitating and/or is a hazard to the subject.

7.1.2. Adverse drug reaction

In the pre-approval clinical experience with a new medicinal product or its new usages, particularly as the therapeutic dose(s) may not be established: all noxious and unintended responses to a medicinal product related to any dose should be considered adverse drug reactions. The phrase responses to a medicinal product means that a causal relationship between a medicinal product and an adverse event is at least a reasonable possibility, i.e. a relationship cannot be ruled out.

7.1.3. Serious adverse event (SAE)

Any untoward medical occurrence that at any dose:

- results in death,
- is life-threatening,
- requires inpatient hospitalization or prolongation of existing hospitalization,
- results in persistent or significant disability/incapacity, or
- is a congenital anomaly/birth defect
- other important medical event

7.1.4. Unexpected and expected adverse events

An unexpected adverse event is an experience not previously reported (in nature, severity or incidence).

Expected reactions reported from previous trials with tadalafil are characterized as follows: Headache, back pain, muscle pain, pain in the limbs, facial flushing, nasal congestion, indigestion and acid reflux, dizziness, stomach pain, blurred vision, eye pain, increased sweating, difficulty breathing, bleeding from the penis, presence of blood in semen and/or urine, palpitations, rapid heartbeat, high blood pressure, low blood pressure, nosebleeds and tinnitus, fainting, seizures and passing memory loss, swelling of the eyelids, red eyes, sudden decrease or loss of hearing, and rash.

7.1.5. Suspected unexpected serious adverse reaction (SUSAR)

An adverse reaction, the nature or severity of which is not consistent with the applicable product information (e.g., Investigator's Brochure for an unapproved investigational product or package insert/summary of product characteristics for an approved product)

7.2. Reporting

7.2.1. Adverse event

Events reported by the subject (in study diaries and/or orally), or reported in answer to an open question by the study nurse/physician, that fall into any of the above definitions must be recorded on the adverse event page of the CRF and should be described in the following manner: For every AE the "start date" and "end date" (and time, if relevant) must be recorded for calculation of the duration of the event. The severity of the event and the frequency of AE episodes should also be assessed. Severity will be recorded as described above (mild, moderate or severe). The relationship to investigational product (likely, possible, unlikely, unknown) will also be recorded as well as the action taken and the outcome. At every visit the subject should also be asked about non-resolved AE(s). If a subject is withdrawn due to an AE this should be followed up until the subject has recovered or the AE has resolved and an outcome assessment has been performed.

Any clinically significant abnormality, including clinical chemistry/hematology results and results from physical examinations, will be recorded as medical history if before the ingestion of the first study tablet and as an AE if obtained after ingestion of the first study tablet.

7.2.2. Adverse drug reaction

All adverse drug reactions will be recorded by the principal investigator.

7.2.3. Serious adverse event (SAE)

The primary investigator and sponsor are the same physical person.

For all events fulfilling the definition of an SAE the investigator must:
- Complete an SAE report
- Forward the AE and SAE reports, along with other supporting documents if applicable, to the sponsor immediately but no later than the end of the next business day.
- All SAEs will also be reported to Lilly by the sponsor.

7.2.4. Suspected unexpected serious adverse reaction (SUSAR)

The primary investigator and sponsor are the same physical person.

The sponsor will comply with the applicable regulatory requirement(s) related to the reporting of SUSARs to the regulatory authority and ensure that the events are registered in the EudraVigilance database and reported to the Ethics Committee. The Swedish MPA assist with reporting of SUSAR to the EudraVigilance database.

All SUSAR will be reported by the sponsor to the Swedish MPA and Ethics Committee. For fatal or life-threatening SUSARs this will be done as soon as possible and in any case no later than seven (7) calendar days after being made aware of the case. If the initial report is incomplete, follow-up information will be submitted within an additional eight (8) days. All other SUSARS will be reported as soon as possible and in any case no later than fifteen (15) calendar days.

7.2.5. Adverse Event Collection Period

All AEs reported from the time of study drug administration until 1 week following the last medication will be collected. In addition, SAEs will be collected from the time the subject signed the study-specific informed consent. Information on SAEs and AEs is collected at each clinic visit.

7.2.6. Annual safety report

As long as the trial is ongoing a safety report will be completed once a year and sent to the Medical Products Agency and the Ethics Committee. The document will define the time period reported and summarize all occurred serious medical events (SAEs). The safety report should also always include a summary assessment of the safety of subjects that are still included in the trial and whether the benefit-risk assessment changed since the study was approved. A copy of this report will also be sent to Lilly.

8. STATISTICS

8.1. Sample size calculation

A power calculation was performed with glucose disposal rate (GDR equals M-value, mg/kg lean body mass/min) in skeletal muscle during glucose clamp as main outcome parameter, with a power of 90% and a significance level of 0.05 according to Altman (28), where N is the number of subjects and SD is the standard deviation for glucose uptake. Based on previous clamp studies in our laboratory, an SD of 0.75 mg/kg/min was found in T2DM patients and a mean difference of 1 mg/kg/min between tadalafil and placebo treatments is anticipated. With these assumptions, 20 T2D patients are sufficient to find statistical differences between the tadalafil and placebo arms.

We assume a drop-out of ca 20% due to side effects (myalgia, dyspepsia, and hypotension) but that should not be a problem because these symptoms are reversible and the primary end point is powered for 20 subjects who finally complete the protocol.

8.2. Populations

8.2.1 Full analysis data set (based on the ITT principle)

The full analysis data set will consist of all randomized subjects with at least one of the main visits visit 4 (week 6) or visit 7 (week 20). The final definition of the full analyses data set will be made at the Clean-file meeting before the database lock.

8.2.2 Per Protocol (PP) population

The PP population will consist of all patients in the full analysis data set without any significant protocol violation. The final definition of the full analyses data set will be made at the Clean-file

meeting before the database lock.

8.2.3 The Safety population

The Safety population will consist of all randomized subject with at least one intake of the study medication. The final definition of the full analyses data set will be made at the Clean-file meeting before the database lock.

8.3. General statistical methodology

Since there is a long period, 14 weeks, between the main outcomes visits at week 6 and week 20 all statistical efficacy analyses will be adjusted for period effects.

For primary efficacy variables and for those secondary efficacy variables where data is obvious non-normal from graphical views, Koch's adaption of Mann-Whitney U-test will be used.

The change from visit 4, last visit in period 1, to visit 7, last visit in period 2 will be calculated for each subject. This change will compared between the subjects with sequence tadalafil 20mg / placebo vs. subjects with sequence placebo / tadalafil 20mg with Mann-Whitney U-test.

For approximately normal distributed secondary efficacy variables a general linear model will be used, with outcome variable as dependent variable and treatment, subject and period as independent class variables. In these analyses above subjects with a missing value at visit 4 and visit 7 will be excluded from the analyses.

All significance analysis will be two-sided and conducted at the 5% significance level.

If the primary efficacy analysis will reach significance the probability mass (5%) will be passed to the first secondary efficacy analysis. If this analysis also reaches significance the probability mass (5%) will be passed to the first secondary efficacy analysis and so on until the first non-significant result. The significant results, if any, in these sequential tests will all be confirmative.

The distributions of the variables will be given as mean, SD, median, minimum and maximum for continuous variables and as number and percentage for categorical variables.

8.4. Efficacy Analyses

8.4.1. Primary efficacy analysis

The primary efficacy analysis will be the analysis of Glucose disposal rate (GDR equals M-value, mg/kg body weight/min during the glucose clamp between Tadalafil 20 mg and Placebo using Koch's adaption of Mann-Whitney U-test on the Full analysis data set at significance level 0.05, see general statistical methodology above.

8.4.2. Secondary efficacy analyses

The secondary efficacy analysis will be the analysis of all secondary efficacy variables given in section 6.2, in that order, between Tadalafil 20 mg and Placebo using the methods given in general statistical methodology above on the Full analysis data set.

Complementary analyses between Tadalafil 20 mg and Placebo will also be performed on the PP population for primary and secondary variables using the same methods as above.

8.5. Demographics and Baseline Characteristics

Demographics and Baseline Characteristics will be tabulated for both Full analysis set and for PP population.

8.6. Safety analyses

All safety analyses will be performed on the safety population. All treatment emergement AEs and SAEs will be coded with MedDRA dictionary and tabulated on PT-level and SOC level.
AEs will also be tabulated by severity and causality.

8.7. Statistical Analysis Plan

A Statistical Analysis Plan (SAP) with all statistical analyses given in detail will be written and signed before database lock.

9. DATA MANAGEMENT

9.1. Recording of data

The investigator will ensure that all data collected in the study are recorded in a timely manner according to any instructions provided.

A Case Report Form (CRF) will be used for data collection. The investigator will ensure that the data are recorded in the CRF as specified in the study protocol and in accordance with the instructions provided. The investigator will sign the completed CRF. A copy of the completed CRF will be archived at the study site. Any changes or corrections to CRFs must be dated, initialled and explained (if necessary) by the investigator or delegated study personnel.

Sections of CRFs should be monitored on a regular basis. Data management and handling will be conducted in accordance with ICH guidelines, scientific and data management principles and SOPs. Research records generated in this study will be stored on a secure electronic database and in locked cabinets. Only authorized personnel will have access to the data.

The PI will archive all study data including relevant correspondence. The documents will be stored in a locked facility at University of Gothenburg. Only the investigators and a research nurse, delegated by the principal investigator will have access to the database and source data. The documents will be retained for at least 10 years after completion of the study.

The investigator and sponsor are the same person. The randomization list will be kept by investigator/sponsor. If needed, the code can be broken via the investigator/sponsor where the code break envelopes will be stored.

10. QUALITY CONTROL AND QUALITY ASSURANCE

10.1. Monitoring

A study monitor will be appointed by the sponsor. The monitor will be appropriately trained and informed about the nature of the study, patient written information, GCP and applicable regulatory requirements. The monitor's qualifications will be documented.

The monitor will have regular contacts with the clinic to verify informed consents of participating subjects and to confirm that:
- facilities remain acceptable
- the investigational team is adhering to the protocol
- data are being accurately recorded in the CRFs
- therapy accountability is being carried out.

The monitor will also ensure source data verification (comparison of the data in the CRF with the medical records and other source data). The extent of monitoring will be defined in a monitoring plan.

Monitoring will be conducted by Gothia Forum, Gröna stråket 12, 413 45 Göteborg (www.gothiaforum.com)

10.2. Audits and inspections

Authorized representatives of the sponsor, a regulatory authority or an Ethics Committee may perform audits or inspection at the centre, including source data verification. The purpose of an audit or inspection is to systematically and independently examine all study-related activities and documents, to determine whether these activities were conducted, and data were recorded, analysed and accurately reported according to the protocol, Good Clinical Practice (GCP) and any applicable regulatory requirements.

10.3. Other

The investigator is responsible for informing all personnel involved about the clinical trial and, if necessary, educate and train all persons participating in the study. This should be checked by the monitor.

11. ETHICS

The study will be conducted in compliance with the protocol, regulatory requirements, Good Clinical Practice and the ethical principles of the Declaration of Helsinki as adopted by the World Medical Assembly, 1964 (and subsequent revisions).

11.1. Informed consent

All study subjects will receive written and verbal information concerning the study prior to any study related procedures. This information will emphasise that participation in the study is voluntary and that the subject may withdraw from the study at any time and for any reason. All subjects will be given the opportunity to ask questions about the study and will be given sufficient time to decide whether or not to participate in the trial. Subjects will consent that data will be

recorded, collected and processed and presented in scientific reports. The data will not identify any persons taking part in the trial, in accordance with the Swedish legislation "Personuppgiftslagen". A copy of the subject information including the signed consent form will be given to the subject. The signed informed consent forms will be filed in the investigator study file.

11.2. Ethics committee

This protocol and any amendments will be submitted to a properly constituted Independent Ethics Committee (IEC) in accordance with ICH guidelines and local legal requirements for formal approval of the study. This means that the protocol will be submitted to the Independent Ethics Committee in the Gothenburg region (IECGR) in Sweden.

11.3. Subject data protection

The Informed Consent Form will incorporate wording that complies with relevant data protection and privacy legislation. Pursuant to this wording, subjects will authorize the collection, use and disclosure of their study data by the investigator and by those persons who need that information for the purposes of the study.

The Informed Consent Form will explain that study data will be stored in a computer database, maintaining confidentiality in accordance with national data legislation. All data computer processed by the sponsor will be identified by Study Code/Subject ID. The Informed Consent Form will also explain that for data verification purposes, authorized representatives of the sponsor, a regulatory authority or an Ethics Committee may require direct access to parts of the hospital or practice records relevant to the study, including subjects' medical history.

11.4. Insurances

The study subjects are covered by the Swedish Patient Injury Act and the Pharmaceutical Insurance.

12. PROTOCOL AMENDMENTS

Any changes in the study protocol will only be effected after written agreement has been obtained between the principal investigator and the sponsor. An amendment to the clinical study protocol will be submitted to the applicable regulatory authorities. Protocol amendments become effective first when the regulatory authorities have provided written approval.

13. REPORT AND PUBLICATIONS

After completion of the study, the results will be analysed and a clinical study report will be prepared. The report will be submitted to the regulatory authorities and will form the basis for one or several manuscript intended for publication in medical journals. A summary of the results of the study will also be published on the internet.

14. STUDY TIMETABLE

Total study length 20 weeks, enrollment period 3-4 years, treatment period 2015-2018. The study is planned to start in May 2015.

14.1. Definition of "End of study"

When last subject has conducted visit 7. Study terminated prematurely if severe side effects occur.

15. LIST OF REFERENCES

1. DeFronzo, R.A., *Insulin resistance, lipotoxicity, type 2 diabetes and atherosclerosis: the missing links. The Claude Bernard Lecture 2009.* Diabetologia, 2010. **53**(7): p. 1270-87.
2. *Effect of intensive blood-glucose control with metformin on complications in overweight patients with type 2 diabetes (UKPDS 34). UK Prospective Diabetes Study (UKPDS) Group.* Lancet, 1998. **352**(9131): p. 854-65.
3. Caballero, A.E., et al., *Microvascular and macrovascular reactivity is reduced in subjects at risk for type 2 diabetes.* Diabetes, 1999. **48**(9): p. 1856-62.
4. Jaap, A.J., et al., *Reduced microvascular hyperaemia in subjects at risk of developing type 2 (non-insulin-dependent) diabetes mellitus.* Diabetologia, 1994. **37**(2): p. 214-6.
5. Serne, E.H., et al., *Microvascular dysfunction: causative role in the association between hypertension, insulin resistance and the metabolic syndrome?* Essays Biochem, 2006. **42**: p. 163-76.
6. Cook, S. and U. Scherrer, *Insulin resistance, a new target for nitric oxide-delivery drugs.* Fundam Clin Pharmacol, 2002. **16**(6): p. 441-53.
7. Mahajan, H., et al., *Vascular and metabolic effects of methacholine in relation to insulin action in muscle.* Diabetologia, 2006. **49**(4): p. 713-23.
8. Murdolo, G., et al., *Effects of Intrabrachial metacholine infusion on muscle capillary recruitment and forearm glucose uptake during physiological hyperinsulinemia in obese, insulin-resistant individuals.* J Clin Endocrinol Metab, 2008. **93**(7): p. 2764-73.
9. Jansson, P.A., et al., *Tadalafil increases muscle capillary recruitment and forearm glucose uptake in women with type 2 diabetes.* Diabetologia, 2010. **53**(10): p. 2205-8.
10. Murdolo, G., et al., *The selective phosphodiesterase-5 inhibitor tadalafil induces microvascular and metabolic effects in type 2 diabetic postmenopausal females.* J Clin Endocrinol Metab, 2013. **98**(1): p. 245-54.
11. Sandqvist, M., et al., *Impaired delivery of insulin to adipose tissue and skeletal muscle in obese women with postprandial hyperglycemia.* J Clin Endocrinol Metab, 2011. **96**(8): p. E1320-4.
12. Aversa, A., et al., *Chronic administration of Sildenafil improves markers of endothelial function in men with Type 2 diabetes.* Diabet Med, 2008. **25**(1): p. 37-44.
13. Hill, K.D., et al., *Phosphodiesterase 5 inhibition improves beta-cell function in metabolic syndrome.* Diabetes Care, 2009. **32**(5): p. 857-9.
14. Hotamisligil, G.S., *Inflammation and metabolic disorders.* Nature, 2006. **444**(7121): p. 860-7.
15. Ijzerman, R.G., et al., *TNF-alpha levels are associated with skin capillary recruitment in humans: a potential explanation for the relationship between TNF-alpha and insulin resistance.* Clin Sci (Lond), 2006. **110**(3): p. 361-8.
16. Hotamisligil, G.S., et al., *Tumor necrosis factor alpha inhibits signaling from the insulin receptor.* Proc Natl Acad Sci U S A, 1994. **91**(11): p. 4854-8.
17. Eringa, E.C., et al., *Physiological concentrations of insulin induce endothelin-dependent vasoconstriction of skeletal muscle resistance arteries in the presence of tumor necrosis*

factor-alpha dependence on c-Jun N-terminal kinase. Arterioscler Thromb Vasc Biol, 2006. **26**(2): p. 274-80.

18. Rosano, G.M., et al., *Chronic treatment with tadalafil improves endothelial function in men with increased cardiovascular risk.* Eur Urol, 2005. **47**(2): p. 214-20; discussion 220-2.
19. Rubinshtein, R., et al., *Assessment of endothelial function by non-invasive peripheral arterial tonometry predicts late cardiovascular adverse events.* Eur Heart J, 2010. **31**(9): p. 1142-8.
20. Wang, H., et al., *Insulin signaling stimulates insulin transport by bovine aortic endothelial cells.* Diabetes, 2008. **57**(3): p. 540-7.
21. Handa, P., et al., *Reduced vascular nitric oxide-cGMP signaling contributes to adipose tissue inflammation during high-fat feeding.* Arterioscler Thromb Vasc Biol, 2011. **31**(12): p. 2827-35.
22. Rizzo, N.O., et al., *Reduced NO-cGMP signaling contributes to vascular inflammation and insulin resistance induced by high-fat feeding.* Arterioscler Thromb Vasc Biol, 2010. **30**(4): p. 758-65.
23. Zhan, S. and D.C. Rockey, *Tumor necrosis factor alpha stimulates endothelin-1 synthesis in rat hepatic stellate cells in hepatic wound healing through a novel IKK/JNK pathway.* Exp Cell Res, 2011. **317**(7): p. 1040-8.
24. Ahren, B., et al., *Clinical measures of islet function: usefulness to characterize defects in diabetes.* Curr Diabetes Rev, 2008. **4**(2): p. 129-45.
25. Gogg, S., U. Smith, and P.A. Jansson, *Increased MAPK activation and impaired insulin signaling in subcutaneous microvascular endothelial cells in type 2 diabetes: the role of endothelin-1.* Diabetes, 2009. **58**(10): p. 2238-45.
26. Jansson, P.A., et al., *A novel cellular marker of insulin resistance and early atherosclerosis in humans is related to impaired fat cell differentiation and low adiponectin.* FASEB J, 2003. **17**(11): p. 1434-40.

16. SIGNATURES

Date: 2015-09-14

Signature (sponsor): [signature]

Name (sponsor): Per-Anders Jansson Prof/ÖL

Statistical Analysis Plan

Final

Product name: Tadalafil

Study Code: MAKROTAD

Effects on insulin resistance with the phosphodiesterase-5 inhibitor tadalafil in type 2 diabetes – a double-blind, placebo-controlled crossover study

2019-12-30

Author

Name/Title:	
Nils-Gunnar Pehrsson / Study Statistician, CEO, Statistiska Konsultgruppen	
Signature: Nils-G. Pehrsson	Date: 9/1-2020

Approvals

Name/Title:	
Per-Anders Jansson / Principal Investigator and Sponsor	
Signature: [signature]	Date: 9/1-2020
Name/Title: Mattias Molin / SAS-Programmer, Statistiska Konsultgruppen	
Signature: [signature]	Date: 20200109

Table of Contents

1 Study Details ... 6
1.1 Study Objectives ... 6
1.2 Overall Study Design ... 7
1.3 Treatment Groups ... 9
1.3 Treatment Groups ... 9
1.4 Sample Size ... 9
1.5 Definition of Study Populations ... 9
1.5.1 Full Analysis Set and Intention-to-Treat (ITT) Population ... 9
1.5.2 Safety Population ... 10
2 Study Variables ... 10
2.1 Baseline Variables ... 10
2.1.1 Demographics and Baseline Characteristics ... 10
2.1.2 Medical History ... 10
2.1.3 Prior and Concomitant Medications ... 10
2.2 Efficacy Variables ... 10
2.2.1 Primary Efficacy Variable ... 10
2.2.2 Secondary Efficacy Variables ... 11
2.2.3 Exploratory Efficacy Variables ... 12
2.3 Safety Variables ... 12
2.3.1 Exposure of Study Drug ... 12
2.3.2 Adverse Events (AEs) ... 12
3 Statistical Methodology ... 12
3.1 General Methodology ... 12
3.2 Patient Disposition and Data Sets Analyzed ... 13
3.3 Protocol Violations/Deviations ... 14
3.4 Demographics and Baseline Characteristics ... 14
3.5 Medical and Surgical History ... 14
3.6 Prior and Concomitant Medications ... 14
3.7 Efficacy Analyses ... 14
3.7.1 Primary Efficacy Analysis ... 14
3.7.2 Secondary Efficacy Analyses ... 15
3.7.3 Exploratory Efficacy Analyses ... 15
3.8 Safety Analyses ... 16
3.8.1 Exposure of Study Drug ... 16

3.8.2 Adverse Events 16
4 Interim Analyses 17
5 Changes of Analysis from Protocol 17
6 Listing of Tables, Figures and Listings 17
6.1 Listing of Tables 17
6.2 Listing of Figures 18
7 References 18

LIST OF ABBREVIATIONS

Abbreviation	Explanation
AE	adverse event
AI	augmentation index
ASA	acid salicylic acid
ATBF	adipose tissue blood flow
ATC	anatomical therapeutic classification
AUC	area under the curve
BMI	body mass index
BP	blood pressure
cGMP	cyclic guanosine monophosphate
CI	confidence interval
CRF	case report form
CRP	c-reactive protein
D-insulin	dialysate insulin
DBP	diastolic blood pressure
DCA	deoxycholic acid
EC_{50}	half maximal effective concentration
ECG	electrocardiogram
EndoPAT	peripheral arterial tonometry
ET-1	endothelin-1
EVF	erythrocyte volume fraction
FAS	full analysis set
FBF	forearm blood flow
FCS	fully conditional specification
FFA	free fatty acids
GDR	glucose disposal rate
HbA1c	hemoglobin A1 c
HDL-C	HDL-cholesterol
HOMA-IR	homeostatic model assessment – insulin resistance
HUVEC	human umbilical vein endothelial cells
IAUC	incremental area under the curve
I-glycerol	interstitial glycerol
I-insulin	interstitial insulin
I-lactate	interstitial lactate
ICAM-1	intercellular adhesion molecule 1
IGF-1	insulin-like growth factor-1
IL-6	interleukin-6
IP-glycerol	interstitial-plasma glycerol difference
IP-lactate	interstitial-plasma lactate difference
IRS	insulin resistance syndrome
ISI	insulin sensitivity index
ITT	intention-to-treat

Iv	intravenous
JNK	c-Jun N-terminal kinase
LBM	lean body mass
LDL-C	LDL-cholesterol
MD	microdialysis
MedDRA	Medical Dictionary for Regualtory Activities
MVEC	microvascular endothelial cells
NO	nitric oxide
o.d.	once daily
P-glycerol	plasma glycerol
P-lactate	plasma lactate
PDE5	phosphodiesterase-5
PKG	cyclic guanosine monophosphate dependent protein kinase
PP	per-protocol
PT	preferred term
RHI	reactive hyperemia index
S-insulin	serum insulin
SAE	serious adverse event
SBP	systolic blood pressure
Sc	subcutaneous
SD	standard deviation
SGLT2	sodium glucose transporter 2
SOC	system organ class
T2D	type 2 diabetes
Tadalafil	PDE5 inhibitor marketed in pill form for treating erectile dysfunction under the name Cialis®
TFLs	tables, figures, and listings
TNF-α	tumor necrosis factor-α
VCAM-1	vascular cell adhesion molecule 1

1 STUDY DETAILS

1.1 Study Objectives

The primary objective of this study is:

- To evaluate the effect of daily administration of 20 mg tadalafil for 6 weeks ("chronic" tadalafil treatment) compared with placebo on insulin sensitivity in muscle by assessing mean glucose disposal rate (mg/kgLBM/min) at 150-180 min during a 3-hour euglycemic hyperinsulinemic glucose clamp (120 mU/m^2/min) in type 2 diabetes (T2D) participants.

The secondary objectives of this study are:

- To evaluate the effect of chronic tadalafil treatment compared with placebo on glucose metabolism measured as ISI, mean blood glucose (HbA1c), circulating fasting glucose concentration, fasting insulin concentration, HOMA-IR and in T2D participants.
- To evaluate the effect of chronic tadalafil treatment compared with placebo on beta-cell function measured as insulin and c-peptide secretion during 30 min after administration of arginine iv in T2D participants.
- To evaluate whether chronic tadalafil treatment compared with placebo down-regulates circulating inflammatory markers including CRP, IL-6 and TNF-alpha in T2D participants.
- To evaluate whether chronic tadalafil treatment compared with placebo increases muscular and sc interstitial insulin, sc interstitial lactate concentrations and more effectively suppresses lipolysis as measured by sc interstitial glycerol concentration during a glucose clamp in T2D participants.
- To evaluate whether chronic tadalafil treatment compared with placebo improves systolic and diastolic blood pressure, FBF, and microvascular endothelial function measured as RHI and albumin creatinine ratio in urine in T2D participants.
- To evaluate chronic tadalafil treatment compared with placebo on circulating markers of liver function, liver steatosis and lipid metabolism including fasting triglycerides, total cholesterol, HDL-C, LDL-C and FFA levels in T2D participants.
- To evaluate chronic tadalafil treatment compared with placebo on body composition including body weight, BMI, waist circumference, relative fat mass, relative fat free mass and sc fat cell diameter in T2D participants.
- To evaluate chronic tadalafil treatment compared with placebo on gene expression in sc adipose tissue in the fasting state and after a 3-hour euglycemic hyperinsulinemic glucose clamp (120 mU/m^2/min) in T2D participants.
- To evaluate chronic tadalafil treatment compared with placebo in exploratory studies on microbiota composition and diversity of the gut microbiota as well as the plasma bile acid profile, in particular the secondary bile acid deoxycholic acid (DCA), in T2D participants.
- To evaluate chronic tadalafil treatment compared with placebo in exploratory studies on cellular signaling in the insulin/IGF-1 and cGMP-PKG-JNK pathways in sc adipocytes and microvascular endothelial cells (MVEC) in T2D participants.

1.2 Overall Study Design

This is a phase II, randomized, double-blind, placebo-controlled crossover study (Fig 1). A crossover design was chosen to reduce the influence of confounding covariates when each subject serves as his or her own control. This design is also statistically efficient and requires fewer subjects. There are seven study visits and the subjects will be included in the study for 20 weeks (Table 1).

Before visits 2, 3, 4, 5, 6 and 7 subjects will be fasting overnight and informed not to take their ordinary medication in the morning. Furthermore, one day prior to a visit (visit 2-7) the participating subjects will not take their study drug and anti-diabetic medication. In addition, medication with ASA will be suspended 1 week before visit 2-7.

The randomization to start with the study drug or placebo will be conducted by Apoteket Produktion & Laboratorier AB (APL, Kungens Kurva, Stockholm). The study drug and the placebo will be taken as tablets and to ensure that the study is double-blind, placebo and tadalafil tablets will be made visually indistinguishable.

Table 1. Activities per visit

Activity	Visit 1, Screening	Visit 2, week 1	Visit 3	Visit 4, week 6	Visit 5, week 14	Visit 6	Visit 7, week 20
Study information	x						
Informed consent	x						
Demographics	x						
Blood sampling (20 ml)	x						
Physical exam	x						
Medical history	x						
Inclusion/exclusion criteria	x						
Blood sampling (fasting, 20-210 ml)	x	x	x	x	x	x	x
Urine sampling	x	x	x	x	x	x	x
Fecal sample		x		x			x
Anthropometry - weight, length, waist, hip	x	x	x	x	x	x	x
ECG	x						
Randomization		x					
EndoPAT		x	x		x	x	
Arginine test			x			x	
Fat biopsy				x			x
Glucose clamp				x			x
Venous occlusion plethysmography				x			x
Xenon clearance				x			x
Subcutaneous and muscle microdialysis				x			x
Body composition analysis (bioimpedance)		x		x	x		x
Concomitant medication		x	x	x	x	x	x
Adverse events		x	x	x	x	x	x
Diary exchange and review			x	x	x	x	x
Study termination							x

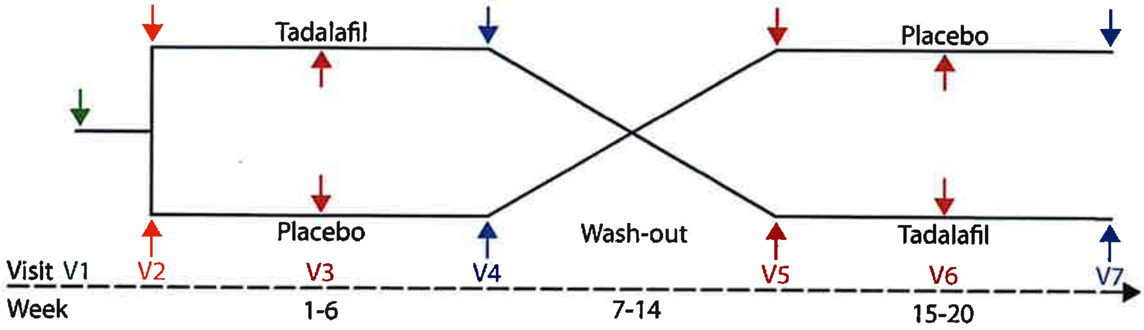

Fig 1. Overview of study protocol. Visit 1: Screening: Study information, informed consent. Anthropometry, ECG, blood and urine sampling. Visit 2: Anthropometry, bioimpedance, blood and urine sampling, fecal collection, EndoPAT, randomization to start treatment. Each treatment period lasts 6 weeks and tadalafil 20 mg or placebo is taken orally o.d. Visit 3 and 6: Anthropometry, blood and urine sampling, EndoPAT as well as an arginine test. Visit 4 and 7: Anthropometry, blood and urine sampling, fecal collection, bioimpedance, glucose clamp, sc needle biopsy procedure before and 3 hours after clamp start, sc and muscle microdialysis, Xenon-clearance and venous occlusion plethysmography. Visit 5: Anthropometry, bioimpedance, blood and urine sampling and EndoPAT.

1.3 Treatment Groups

In this crossover study participants will receive either Cialis® or placebo in treatment period I, and the respective remaining treatment in treatment period II:

- Cialis® (active substance tadalafil; called Tadalafil in the TFLs): daily administration of 20 mg tadalafil in tablets for 6 weeks.
- Placebo: daily administration of tablets for 6 weeks.

1.4 Sample Size

A power calculation was performed with glucose disposal rate (GDR equals M-value, mg/kg LBM/min) in skeletal muscle during glucose clamp as main outcome parameter, with a power of 90% and a significance level of 0.05 according to Altman (28), where N is the number of subjects and SD is the standard deviation for glucose uptake. Based on previous clamp studies in our laboratory, an SD of 1.50 mg/kgLBM/min was found for differences in T2D patients and a mean difference of 1 mg/kgLBM/min between tadalafil and placebo treatments is anticipated. With Fisher's permutations test for paired observations and these assumptions, 20 T2D patients in total are sufficient to find statistical differences between the tadalafil and placebo arms.

We assume a drop-out rate of ca 20% due to side effects (myalgia, dyspepsia, and hypotension) but that should not be a problem because these symptoms are reversible and the primary end point is powered for 20 participants who finally complete the protocol. Therefore, we aimed to randomize 25 T2D participants.

1.5 Definition of Study Populations

1.5.1 Full Analysis Set and Intention-to-Treat (ITT) Population

The full analysis set (FAS) will consist of all randomized subjects with both main visits, i.e. visit 4 (week 6) and visit 7 (week 20).

Sensitivity analyses for the primary efficacy variable and the two first secondary variables will be performed on the ITT-population with all randomized patients with no less than a completed main visit 4, using multiple imputation.

Per-Protocol (PP) Population

The PP population will consist of all patients in the FAS without any significant protocol violation. The final definition of the PP data set will be made at the Clean-file meeting before the database lock.

1.5.2 Safety Population

The safety population will consist of all randomized subjects with at least one intake of the study medication. Study Variables

2 STUDY VARIABLES

2.1 Baseline Variables

2.1.1 Demographics and Baseline Characteristics

Gender, age, BMI, waist, hip, systolic blood pressure, diastolic blood pressure, diabetes duration, T2D heredity (1st degree), current smoker (n, %), current smokeless tobacco (n, %), fP-Glucose, fS-Insulin, HOMA-IR, B-HbA1c, S-Cholesterol, S-LDL-C, S-HDL-C, S-Triglycerides, U-Albumin/Creatinine ratio, S-Creatinine, S-ALT, S-AST, S-C-reactive protein; pharmacological diabetes treatment (n, %; metformin, sulfounylurea, insulin or SGLT2-inhibitors), anti-hypertensive treatment (n, %; calcium channel blocker, thiazides or loop diuretics), treatment for hypercholesterolemia with statins (n, %; simvastatin, atorvastatin or rosuvastatin), ASA, microvascular complications (n, %; retinopathy, nephropathy or neuropathy).

2.1.2 Medical History

At screening physical examination and interview of medical history will be performed by a specialist in diabetology (Per-Anders Jansson, PI, Prof). Diseases will be coded and classified in a supplemental table.

2.1.3 Prior and Concomitant Medications

Prior medication is defined as pharmacological treatment at screening visit and concomitant medication is prior medication and all changes of pharmacological treatment from the screening visit and throughout the study. Concomitant medication is classified and coded according to higher level anatomical therapeutic classification (ATC) group and generic term.

2.2 Efficacy Variables

2.2.1 Primary Efficacy Variable

Primary efficacy variable is GDR (which equals M-value, mg/kgLBM/min) at steady state (150-180 min) during the glucose clamp. The variable is measured after 6 weeks of treatment in each treatment period. A comparison will be made between the M-value in treatment period I (visit 4) and treatment period II (visit 7).

2.2.2 *Secondary Efficacy Variables*

Regarding change from baseline to 3 and 6 weeks, baseline will be defined as measurements at visit 2 and visit 5 if done (baseline for treatment period I and II, respectively), otherwise baseline will be the last observation done at visit 1 and/or visit 2.

Secondary efficacy variables are:

- Insulin sensitivity index (ISI) at clamp steady state.
- Mean blood glucose (HbA1c).
- Insulin resistance markers (Fp-glucose, fs-insulin, HOMA-IR).
- Beta-cell function (IAUC glucose 0-30 min, IAUC insulin 0-30 min, IAUC insulin/glucose ratio 0-30 min, IAUC C-peptide 0-30 min, IAUC C-peptide/glucose 0-30 min, IAUC insulin 0-10 min, IAUC insulin/glucose ratio 0-10 min, IAUC C-peptide 0-10 min, IAUC C-peptide/glucose 0-10 min, delta value – baseline level to peak value for insulin, delta value – baseline level to peak value for C-peptide).
- Inflammatory markers (s-Endothelin-1, s-IL-6, cellular adhesion molecules (ICAM, VCAM, E-Selectin) and hs-CRP).
- Muscle and sc microdialysis insulin levels (Insulin levels at baseline, mean of 2-4 measurements for muscle d-Insulin and sc d-Insulin; delta value -muscle d-Insulin and sc d-Insulin at baseline vs 180 min, absolute and relative changes of insulin levels are compared; muscle d-Insulin and sc d-Insulin and time to reach EC_{50} at clamp steady state and mean of muscle d-Insulin and sc d-Insulin concentration of these variables at 150 -180 min; AUC muscle d-insulin and AUC sc d-Insulin 0-180 min).
- Hemodynamic variables (Baseline FBF, IAUC FBF 0-180 min, systolic BP, diastolic BP, pulse rate, RHI, post-occlusion/baseline signal ratio during 5 min).
- Lipolysis markers (At baseline, mean of 2-4 measurements for P-Glycerol and sc I-Glycerol; delta value - P-Glycerol and sc I-Glycerol at baseline vs mean 150 - 180 min and relative change of glycerol levels are compared; P-Glycerol, sc I-Glycerol and IP-Glycerol mean concentration at 150 -180 min as well as time to reach EC_{50} of these variables at clamp steady state; AUC sc I-Glycerol and AUC P-Glycerol 0-180 min).
- Glycolysis markers (At baseline, mean of 2-4 measurements for P-Lactate, sc I-Lactate and sc I-Glucose; delta value - P-Lactate, sc I-Lactate and sc I-Glucose at baseline vs mean 150 - 180 min, absolute and relative changes of lactate and glucose levels are compared; P-Lactate, sc I-Lactate, sc I-Glucose, IP-Glucose, IP-Lactate and time to reach EC_{50} at clamp steady state and mean of concentration of these variables at 150 -180 min; AUC sc I-Lactate, AUC sc I-Glucose and AUC P-Lactate 0-180 min.
- Adipose tissue gene expression (fasting state and change during the glucose clamp).
- Anthropometric measures (Body weight, waist, hip, waist/hip ratio, BMI, body fat (kg), body fat (% of body weight), LBM (kg), LBM (% of body weight) and fat cell size).
- Circulating markers of lipid metabolism (P-FFA, S-Triglycerides, S-Cholesterol, S-HDL-C and S-LDL-C).
- Other laboratory measurements (U-Albumin/Creatinine ratio, S-ALT, S-AST, S-ALP, S-Bilirubin, S-Hb, S-Na, S-K, S-Ca, S-Creatinine and EVF).

2.2.3 Exploratory Efficacy Variables

Comparisons are made between microbiota composition and diversity of the gut microbiota and plasma bile acid profile in the fasting state and after 6 weeks in treatment period I (visit 4) and after 6 weeks in treatment period II (visit 7).

Comparisons are made between explorative urine protein analyses in the fasting state and after 6 weeks in treatment period I (visit 4) and after 6 weeks in treatment period II (visit 7).

Comparisons are made between exploratory studies on cellular signaling in the insulin/IGF-1 and cGMP-PKG-JNK pathways in sc adipocytes after 6 weeks in treatment period I (visit 4) and after 6 weeks in treatment period II (visit 7).

2.3 Safety Variables

2.3.1 Exposure of Study Drug

Compliance will be defined by counting tablets in returned bottles at the end of each treatment period aiming for > 80% exposure of study drug during treatment periods I and II.

2.3.2 Adverse Events (AEs)

The investigator will monitor each subject for evidence of AEs on a routine basis throughout the study. The investigator will assess and record any AE in detail including the date of onset, description, severity, duration and outcome, relationship of the AE to study drug, and any action(s) taken. AEs, whether in response to a query, observed by site personnel, or reported spontaneously by the subject, will be recorded. All AEs will be followed until a satisfactory conclusion. Causality, severity, start date, end date and if serious is given for each AE.

Classification according to the Medical Dictionary for Regulatory Activities (MedDRA) terminology will be presented in the Study Report. Safety data will be listed and summarized and presented descriptively and presented in tabular and/or graphical form. All AE data will be coded and listed individually and summarized using MedDRA terminology.

3 STATISTICAL METHODOLOGY

3.1 General Methodology

The distributions of the variables will be given as mean, SD, median, minimum and maximum for continuous variables and as number and percentage for categorical variables.

Since there is a long period, 14 weeks, between the final visits of treatment period I (week 6) and treatment period II (week 20), all statistical efficacy analyses will be adjusted for period effects.

For the following three variables in the following order, a fixed-sequence test will be performed between the tadalafil and placebo groups:

- primary efficacy variable, GDR at steady state during the glucose clamp.
- first secondary efficacy variable, ISI at clamp steady state.
- second secondary efficacy variable, mean blood glucose (HbA1c) change from baseline to 6 weeks treatment.

If the primary efficacy variable is shown to be significant at 5% significance level, the probability mass 5% will be transferred to the first secondary efficacy variable. If this also will be shown to be significant, the probability mass 5% will be transferred to the second

secondary variable. When the first of these three tests is non-significant only the significant test above will be confirmatory. If all three tests will be significant at 5% significance level all three will be confirmative. These analyses will be performed primary on the FAS population se methods below.

For these three variables, the following sensitivity analyses will also be performed: missing data will be imputed using multiple imputation by fully conditional specification (FCS) (SEED = 7739) with 50 imputation datasets. Each of these 50 datasets will be analysed with a general linear model with outcome variable as dependent variable and treatment, subject and period as independent class variables. A pooled analysis over the distributions of these test variables will give the p-value and mean difference between the groups with 95% confidence interval.

Primary analysis and all secondary efficacy analyses will be performed on the FAS population on available data in the following way:

- For obvious non-normal from graphical views, **Fisher's non-parametric two-sample permutation test** will be used in the following way for the analysis between tadalafil 20mg and placebo:

  The change from the M-value in treatment period I to the value in treatment period II will be calculated for each subject. This change will be compared between the subjects with sequence tadalafil 20mg / placebo vs. subjects with sequence placebo / tadalafil 20mg with **Fisher's non-parametric two-sample permutation test** (1).

- For approximately normal distributed secondary efficacy variables a general linear model will be used, with outcome variable as dependent variable and treatment, subject and period as independent class variables.

- For dichotomous and ordered categorical variables the change from treatment period I to treatment period II will be calculated as increase, no change, or decrease. This ordered categorical changes will then be compared between the subjects with sequence tadalafil 20mg / placebo vs. subjects with sequence placebo / tadalafil 20mg with Mantel-Haenszel chi-square test.

In these analyses on the FAS population on available data, subjects with missing values in treatment period I or II will be excluded from the analyses comparing the placebo and tadalafil treatment periods.

For all analyses of continuous variables between tadalafil and placebo mean difference with 95% confidence interval (CI) will be given based on Fisher's non-parametric permutation test.

All the analyses of primary and secondary efficacy variables between tadalafil 20 mg and placebo will be analyzed on the FAS and primary and selected secondary efficacy variables will be analyzed on the PP population.

For analyses of change within treatment, Fisher's non-parametric permutation test for paired observations will be used for continuous variables and Sign test for dichotomous and ordered categorical variables combining the results for both treatment periods. Mean change with 95% CI will be given for analysis of change of continuous variables within the two treatments.

All significance tests will be two-tailed and conducted at 0.05 significance level. All analyses will be performed using SAS® v9.2 (Cary, NC, USA).

3.2 Patient Disposition and Data Sets Analyzed

The number of subjects included in each of the ITT, PP and safety populations will be summarized. The number and percentage of subjects randomized and treated will be

presented. Subjects who completed the study and subjects who withdrew from the study prematurely will also be presented with a breakdown of the reasons for withdrawal by treatment group for the ITT, PP and safety populations.

3.3 Protocol Violations/Deviations

Major protocol deviations are those that are considered to have an effect on the analysis. A list of potential major protocol deviations will be generated programmatically from the data captured before the Clean-file meeting. The clinical monitors of the study will review the list and the finalization of the major protocol deviations will be done at the Clean-file meeting.

The number of patients with major protocol deviations will be summarized per treatment group.

3.4 Demographics and Baseline Characteristics

Demographics and baseline characteristics will be summarized for the ITT and PP populations.

3.5 Medical and Surgical History

Medical history will be summarized by system organ class (SOC) and preferred term (PT) for the total FAS population.

3.6 Prior and Concomitant Medications

Prior and concomitant medication will be summarized by higher level ATC group and generic term for each treatment period for FAS population. Concomitant medication and any changes in existing medication during the study will be recorded in the case report form (CRF) and reported in the Study Report. The medication will be classified by the active ingredient according to the ATC classification system. Participants are allowed to continue with their regular medications during the study, if not otherwise indicated.

3.7 Efficacy Analyses

3.7.1 Primary Efficacy Analysis

For primary efficacy variable, glucose disposal rate (GDR equals M-value, mg/kgLBM/min) at steady state (150-180 min) during the glucose clamp Fisher's non-parametric permutation test will be used in the following way for the analysis between tadalafil 20mg and Placebo:

- The change from the value in period 1 to the value in period 2 will be calculated for each subject. This change will compared between the subjects with sequence tadalafil 20mg / placebo vs. subjects with sequence placebo / tadalafil 20mg with two-sided Fisher's non-parametric two-sample permutation test on the full analysis set at significance level 0.05.
- The estimated mean difference with 95% CI between tadalafil 20mg and Placebo will be given based on the permutation test. The effect will also be visualised graphically.

The following sensitivity analyses will also be performed on the ITT population: missing data will be imputed using multiple imputation by fully conditional specification (FCS) (SEED = 7739) with 50 imputation datasets. Each of these 50 datasets will be analysed with a general linear model with primary efficacy variable as dependent variable and

treatment, subject and period as independent class variables. A pooled analysis over the distributions of these test variables will give the p-value and mean difference between the groups with 95% confidence interval.

A sensitivity analysis using the same nonparametric will also be performed on the PP-population.

3.7.2 Secondary Efficacy Analyses

All secondary efficacy variables given in section 2.2.2 will be analyzed according to the statistical methods given in section 3.1 General methodology.

All secondary efficacy analysis will be two-sided on the FAS and conducted at the 5% significance level. Secondary efficacy analyses will also be performed on the PP population.

3.7.3 Exploratory Efficacy Analyses

The following exploratory efficacy analyses will be done:

- Extraction of fecal genomic DNA

Total genomic DNA will be isolated from 100 mg of feces using a repeated bead beating method based on a protocol previously described. Briefly, fecal samples are placed in Lysing Matrix E tubes (MP Biomedicals) and extracted twice in lysis buffer (4% w/v SDS; 500 mmol/L NaCl; 50 mmol/L EDTA; 50 mmol/L Tris-HCl; pH 8) with bead beating at 5.5 m/s for 45 s in a FastPrep®-24 Instrument (MP Biomedicals) (2).

- Fecal microbiota composition

The fecal microbiota composition will be profiled in matching fecal samples from patients at baseline, visit 4 and visit 7 by sequencing the V4 region of the 16S rRNA gene on an Illumina MiSeq instrument (llumina RTA v1.17.28; MCS v2.5) with the V2 Illumina kit (2x250 bp paired-end reads). Only matching samples from the same individual collected at these time points and sequenced at a depth higher than 20,000 sequences/sample shall be included in downstream analyses of microbiota diversity (3).

- Analysis of plasma bile acids

Bile acids will be measured in 25 µL of homogenized plasma (taken at baseline, visit 4 and visit 7) added to methanol (250 µl) containing 50 nM of the internal standards D4-cholate, D4-chenodeoxycholate, D4-glycocholate, D4-taurocholate, D4-glycochenodeoxycholate and D4-taurochenodeoxycholate. Subsequently, samples will be mixed and centrifuged for 10 min at 20000g. The supernatant will then be removed, evaporated under a stream of nitrogen at 40°C and reconstituted in 100 µL of 50% methanol. Bile acids will be measured using liquid chromatography tandem mass spectrometry (LC-MS/MS) as described previously (3). Only pairwise data will be presented.

- Gene expression and cell signaling in adipose tissue

Subcutaneous adipose tissue biopsies will be obtained and for studies of cellular signaling adipocytes will be isolated with collagenase ~~as previously~~ reported (4). Furthermore, adipose tissue is snap-frozen for measurement of gene expression and immunoblot analysis.

- Real-time PCR

Total RNA will be isolated using the RNeasy Lipid Tissue mini kit (Qiagen, Hilden, Germany). Reagents for genes of interest for real-time PCR shall be purchased from Applied Biosystems and used according to the manufacturer's protocol. The gene expression levels will be normalized to the housekeeping gene 18S rRNA (Applied Biosystems) (4).

- Immunoblotting

Sc adipose tissue will be homogenized in ice-cold lysate buffer with Complete Protease Inhibitor Cocktail (Roche Applied Science, Mannheim, Germany), and lysed at 4°C for 90 min. The supernatant, a fat-free extract, will be obtained by centrifugation at 12 000x*g*, 15 min, 4°C. Protein concentration will be measured with the BCA protein assay kit (Thermo Scientific, Rockford, IL, USA). The lysate will be subjected to SDS-PAGE, transferred to a nitrocellulose membrane and immunoblotted with an antibody for the protein of interest (Millipore, San Diego, CA, USA) and thereafter with a secondary antibody (Cell Signaling Technologies, Beverly, MA, USA). A loading control shall be used and detection shall be performed using a ChemiDoc XRS detection system (Bio-Rad) (4).

- Mass spectrometry

The proteome of biobanked urine will be studied essentially as previously described (5).

3.8 Safety Analyses

3.8.1 Exposure of Study Drug

Duration of therapy will be summarized for each treatment. Compliance will be summarized for each treatment at the visits at 3 weeks and 6 weeks from the start of each treatment period.

The summaries will be provided for the safety population.

3.8.2 Adverse Events

Only treatment-emergent AEs compared to baseline visit in the first treatment period will be included in the summaries for the safety population.

A summary of subjects reporting at least one of the following AEs will be presented in an overview table:

- Any AE
- Any serious AE (SAE)
- Any treatment-related AE
- Any treatment-related SAE
- Any AE leading to discontinuation

Summaries per SOC and PT presenting n (%) of AEs and n (%) of subjects with at least one AE will be provided for:

- All AEs (includes all serious and non-serious AEs)
- All AEs by maximum reported intensity
- All AEs by causality
- All SAEs
- All AEs leading to discontinuation.

4 INTERIM ANALYSES

No interim analyses will be performed.

5 CHANGES OF ANALYSIS FROM PROTOCOL

Information about the secondary variables has been given in detail and some secondary variables have been added. For primary analyses and some secondary efficacy analyses a multiple imputation with 50 imputation datasets will be used. Moreover, missing results of the interstitial measurements in sc adipose tissue and skeletal muscle will be substituted by linear imputation. Comparison between treatments for the primary outcome will be tested using a general linear model with outcome variable as dependent variable and tadalafil 20mg / placebo, subject and period as independent class variables with multiple imputation and using Koch's adaption of Fisher's non-parametric two-sample permutation test instead of Koch's adaption of the Mann-Whitney U-test. This enables presentation of the results with 95% CI. Our estimate of SD for the primary efficacy variable has been suggested to be 1.50 mg/kgLBM/min and not 0.75 mg/kgLBM/min as we anticipated when we wrote the protocol.

6 LISTING OF TABLES, FIGURES AND LISTINGS

6.1 Listing of Tables

Table Number	Table Title
14.1.1	Patient Disposition and Data Sets Analyzed (ITT Population)
14.1.2	Protocol Deviations Leading to Exclusion from PP Population (ITT Population)
14.1.3.1	Demographics and Baseline Characteristics (ITT Population)
14.1.3.2	Demographics and Baseline Characteristics (PP Population)
14.1.4	Medical History (ITT Population)
14.1.5.1	Prior Medications (ITT population)
14.1.5.2	Concomitant Medications (ITT population)
14.2.1.1	Primary Efficacy Analysis (FAS Population)
14.2.1.2	Primary Efficacy Analysis, sensitivity analysis (ITT Population)
14.2.1.3	Primary Efficacy Analysis, sensitivity analysis (PP Population)
14.2.2.1	Secondary Efficacy Analyses (FAS Population)
14.2.2.2	Two Selected Secondary Efficacy Analyses (ITT Population)
14.2.2.3	Selected Secondary Efficacy Analyses (PP Population)
14.3.1.1	Duration of Exposure (Safety Population)
14.3.1.2	Compliance (Safety Population)
14.3.2.1	Summary of Adverse Events (Safety Population)
14.3.2.2	Adverse Events, by System Organ Class and Preferred Term (Safety Population)
14.3.2.3	Adverse Events, by System Organ Class, Preferred Term and Maximum Reported Intensity (Safety Population)
14.3.2.4	Adverse Events, by System Organ Class, Preferred Term and Causality Assessment (Safety Population)
14.3.2.5	Serious Adverse Events, by System Organ Class and Preferred Term (Safety Population)
14.3.3	Adverse Events Leading to Discontinuation, by System Organ Class and Preferred Term (Safety Population)

6.2 Listing of Figures

Figure Number	Figure Title
14.1.1	Primary Efficacy Analysis (FAS population)
14.1.2	Primary Efficacy Analysis, sensitivity analysis (ITT Population)
14.1.3	Primary Efficacy Analysis, sensitivity analysis (PP Population)
14.2.1-x	Secondary Efficacy Analyses (FAS population)
14.2.2-x	Two Selected Secondary Efficacy Analyses (ITT Population)
14.2.3-x	Selected Secondary Efficacy Analyses (PP population)
16.2.1	Discontinued Patients
16.2.2	Patients with Important Protocol Deviations
16.2.3	Patients Excluded from the Efficacy Analysis
16.2.4.1	Demographics and Baseline Characteristics
16.2.4.2	Medical History
16.2.4.3	Surgical History
16.2.4.4	Prior and Concomitant Medications
16.2.5	Compliance and Drug Exposure
16.2.6	Efficacy Variables
16.2.7	Adverse Events
16.2.8	Laboratory Data
16.2.9	Vital Signs Data
16.2.10	ECG Data
16.2.11	Abnormal Physical Examination Data

7 REFERENCES

1. Stephen Senn,"Cross-over Trials in Clinical research, second edition", John Wiley & Sons Ltd, 2002
2. Salonen A, Nikkila J, Jalanka-Tuovinen J, Immonen O, Rajilic-Stojanovic M, Kekkonen RA, Palva A, de Vos WM: Comparative analysis of fecal DNA extraction methods with phylogenetic microarray: effective recovery of bacterial and archaeal DNA using mechanical cell lysis. J Microbiol Methods 2010;81:127-134
3. Tremaroli V, Karlsson F, Werling M, Stahlman M, Kovatcheva-Datchary P, Olbers T, Fandriks L, le Roux CW, Nielsen J, Backhed F: Roux-en-Y Gastric Bypass and Vertical Banded Gastroplasty Induce Long-Term Changes on the Human Gut Microbiome Contributing to Fat Mass Regulation. Cell metabolism 2015;22:228-238
4. Jansson PA, Pellmé F, Hammarstedt A, Sandqvist M, Brekke H, Caidahl K, Forsberg M, Volkmann R, Carvalho E, Funahashi T, Matsuzawa Y, Wiklund O, Yang X, Taskinen MR, Smith U. A novel cellular marker of insulin resistance and early atherosclerosis in humans is related to impaired fat cell differentiation and low adiponectin. FASEB J. 2003;17:1434-40.
5. Adachi J, Kumar C, Zhang Y, Olsen J and Mann M. The human urinary proteome contains more than 1500 proteins including a large proportion of membrane proteins. Genome Biology 2006;7 (9):R80
