# Supplementary material for: Feasibility of high-dose tadalafil and effects on insulin resistance in well-controlled patients with type 2 diabetes (MAKROTAD): a single-centre, double-blind, randomised, placebo-controlled, cross-over phase 2 trial
Source: eClinicalMedicine. 2023 May 4;59:101985. doi: 10.1016/j.eclinm.2023.101985 (PMC10225663; doi:10.1016/j.eclinm.2023.101985)
Supplement: Translated abstract [file mmc2.doc]

**The following translation in Swedish was submitted by the authors and we reproduce it as supplied. It has not been peer reviewed. Our editorial processes have only been applied to the original abstract in English, which should serve as reference for this manuscript**

**Abstrakt**

**Bakgrund** Fosfodiesteras-5-hämmare uppvisar positiva effekter på blodkärl och ämnesomsättning hos patienter med typ 2 diabetes (T2D) men om läkemedelsgruppen har effekt på insulinresistens hos patienter med T2D är inte känt.

**Metoder** En randomiserad, dubbel-blind, placebo-kontrollerad, cross-over studie genomfördes på Sahlgrenska Universitetssjukhuset i Göteborg. Män utan problem med erektil dysfunktion (ålder 40-70 år) och kvinnor (ålder 55-70 år, postmenopausala), duration av T2D mellan 3 månader – 10 år, hemoglobin A1c (HbA1c) <60 mmol/mol och med BMI 27-40 kg/m^2^ inkluderades. Deltagarna randomiserades till en period med oralt intag av tadalafil 20 mg och en period med placebo, en gång om dagen i 6 veckor, separerade av en washout period på 8 veckor. Tabletter med placebo och tadalafil gick ej att skilja åt för ögat och levererades randomiserat i varsin kartong till studien från Apoteket Produktion & Laboratorier AB, Stockholm. Efter inklusion tilldelades deltagarna ett studienummer och randomiserades till motsvarande studieläkemedel för period A och period B. Varje behandlingsperiod avslutades med en glukos-clamp och mätningar av kroppssamman-sättning, mätningar av metabola markörer i blodprover och mätningar i vävnadsdialysat från subkutan fettväv och muskel. Primär målsättning var att jämföra insulinkänslighet mätt med glukos-clamp efter 6 veckors behandling i respektive studieperiod och sekundära målsättningar var att studera effekter av tadalafil avseende patofysiologi vid T2D och hur väl deltagare tolererar en hög dos av tadalafil. Den primära analysen gjordes på deltagare med kompletta besök och säkerhetsanalysen utfördes på samtliga deltagare som tagit minst en dos av studieläkemedlet. Studien är registrerad i databaserna ClinicalTrials.gov (NCT02601989) och EudraCT (2015-000573).

**Resultat** Mellan 22 januari 2016 och 31 januari 2019 inkluderades 23 deltagare med T2D av vilka 18 deltagare hade kompletta besök. Vi fann att effekten av tadalafil på insulinresistens, mätt med clamp, inte skiljde sig jämfört placebo. Emellertid minskade tadalafil medelblod-socker uppmätt med HbA1c (medelvärde för skillnad mellan behandlingsperioderna -2,50 mmol/mol, 95% konfidensinsintervall, -4,20; -0,78, p=0,005). Vidare sågs en förbättring av endotelfunktion och markörer för leverförfettning och glykolys, medan ingen statistiskt signifikant skillnad mellan behandlingarna påvisades för annan utförd karakteristik på deltagarna. Muskelvärk, dyspepsi och huvudvärk förekom oftare hos deltagare som fick högdos tadalafil jämfört placebo (p <0,05) men allvarliga biverkningar skilde sig inte mellan behandlingarna.

**Tolkning** Högdos tadalafil minskar inte insulinresistens mätt med clamp, men förbättrar endotelfunktion och markörer för leverförfettning och ämnesomsättning, parallellt med en optimering av metabol kontroll uppmätt med HbA1c. Högdos tadalafil tolereras i varierande grad varför större studier bör initieras för att komma fram till rätt anpassad dosering för patienter med T2D.
